# Adverse drug reactions in older adults: a retrospective comparative analysis of spontaneous reports to the German Federal Institute for Drugs and Medical Devices

**DOI:** 10.1186/s40360-020-0392-9

**Published:** 2020-03-23

**Authors:** Diana Dubrall, Katja S. Just, Matthias Schmid, Julia C. Stingl, Bernhardt Sachs

**Affiliations:** 1 Institute for Medical Biometry, Informatics and Epidemiology (IMBIE), University Hospital of Bonn, Bonn, North Rhine-Westphalia Germany; 2 Research Division, Federal Institute for Drugs and Medical Devices (BfArM), Bonn, North Rhine-Westphalia Germany; 3 Institute of Clinical Pharmacology, University Hospital of the RWTH Aachen, Aachen, North Rhine-Westphalia Germany; 4 Department for Dermatology and Allergy, University Hospital of the RWTH Aachen, Aachen, North Rhine-Westphalia Germany

**Keywords:** Adverse drug reactions, Spontaneous reports, ADR database, Adverse drug reactions older adults, Side effects, Older adults

## Abstract

**Background:**

Older adults are more prone to develop adverse drug reactions (ADRs) since they exhibit numerous risk factors. The first aim was to analyse the number of spontaneous ADR reports regarding older adults (> 65) in the ADR database of the German Federal Institute for Drugs and Medical Devices (BfArM) and to set them in relation to i) the number of ADR reports concerning younger adults (19–65), and ii) the number of inhabitants and assumed drug-exposed inhabitants. The second aim was to analyse, if reported characteristics occurred more often in older vs. younger adults.

**Methods:**

All spontaneous ADR reports involving older or younger adults within the period 01/01/2000–10/31/2017 were identified in the ADR database. Ratios concerning the number of ADR reports/number of inhabitants and ADR reports/drug-exposed inhabitants were calculated. The reports for older (*n* = 69,914) and younger adults (*n* = 111,463) were compared using descriptive and inferential statistics.

**Results:**

The absolute number of ADR reports involving older adults increased from 1615 (2000) up to 5367 ADR reports (2016). The age groups 76–84 and 70–79 had the highest number of ADR reports with 25 ADR reports per 100,000 inhabitants and 27 ADR reports per 100,000 assumed drug-exposed inhabitants. For both ratios, the number of reports was higher for males (26 and 28 ADR reports) than for females (24 and 26 ADR reports). Fatal outcome was reported almost three times more often in older vs. younger adults. Six out of ten drug substances most frequently suspected were antithrombotics (vs. 1/10 in younger adults). For some drug substances (e.g. rivaroxaban) the ADRs reported most frequently differed between older (epistaxis) and younger adults (menorrhagia).

**Conclusions:**

There is a need to further investigate ADRs in older adults since they occurred more frequently in older vs. younger adults and will likely increase in future. Physicians should be aware of different ADRs being attributed to the same drug substances which may be more prominent in older adults. Regular monitoring of older adults taking antithrombotics is recommended.

## Background

Older adults usually present with many risk factors promoting the occurrence of adverse drug reactions (ADRs) [1] like e.g. multimorbidity which can lead to polypharmacy [2]. In Germany, up to 58% of older adults suffer from at least one chronic disease [3], and around 50% in the age group of 70–79 years exhibit polypharmacy [4]. Further risk factors for ADRs in older adults include changes in renal and hepatic clearance, distribution and metabolism leading to prolonged half-lives or higher plasma concentrations if not taken into consideration [5].

With regard to spontaneously reported ADRs roughly three times more ADR reports per million inhabitants per year are reported for older adults aged 65–74 years compared to younger adults aged 5–19 years for high-income countries [6]. Since ADRs are an important cause for morbidity and death [7], they have a significant impact on healthcare systems, especially in older adults [8]. For example, ADR-related hospital admissions are more common in older than younger adults in two German observational studies [9, 10]. Concerning ADRs resulting in death, the highest number of reported fatal ADRs is reported for the older adults aged 71–80 years in a Swedish study [11].

Since the proportion of older adults within the German population is steadily increasing [12] (in 2060 roughly every third person will be ≥65 years [13]) the impact and significance of ADRs in older adults is supposed to gain further medical and economic relevance in the future.

In general, ADRs in older adults may be difficult to recognise as they often present with unspecific symptoms or are attributed to underlying diseases. Therefore, the causal association with drug treatment is difficult to assess [10, 14] and the prevalence of ADRs in older adults might even be higher. With regard to the reporting of ADRs, some (older) studies found that ADRs in older adults are less often reported [15, 16] whereas a recent study describes the opposite [17].

Since some drugs were found to be associated more often with ADRs in older adults, lists of potentially inappropriate medications (PIMs) for older adults (e.g. PRISCUS list, international Beers Criteria) have been published [18–20]. Irrespective of these lists of PIMs, in spontaneous reports from Italy and Sweden the drug classes reported most frequently to be associated with ADRs in older adults are cardiovascular drugs and drugs acting on the blood and blood forming organs [17, 21].

The present study is the first retrospective analysis of spontaneous ADR reports (specified as “ADR reports” in the following) concerning older adults (> 65 years) performed in the large ADR database of the Federal Institute for Drugs and Medical Devices (BfArM) [22]. The first aim of the study was to determine the number of ADR reports regarding older adults (> 65 years) and to set these reports in relation to i) the number of spontaneous ADR reports regarding younger adults (19–65), and ii) the number of inhabitants [23] and assumed drug-exposed inhabitants [4], and to oppose the ADR reports to the number of defined daily doses (DDD) used per insured person [24]. The second aim was to analyse, if some of the reported characteristics are more often described in the ADR reports of older adults compared to younger adults.

## Methods

### Reporting channels

Physicians in Germany are obliged by their professional code of conduct to report ADRs to their professional councils which forward these reports to either BfArM (responsible for chemically defined drugs) or Paul-Ehrlich-Institut (PEI) (responsible for monoclonal antibodies, vaccines etc.) as described elsewhere [25]. BfArM and PEI are independent federal higher authorities within the portfolio of the Federal Ministry of Health (so called competent authorities) [26].

Both, Health Care Professionals (HCPs) and Non-Health Care Professionals (non-HCPs, e.g. consumer) may also directly report to one of these two competent authorities, or to the respective marketing authorization holders.

ADRs can be reported online [27, 28] or by using standardized reporting forms. Alternatively a reporting by fax, scan, or postal mail, or directly (without a form) by postal mail, fax, or email is also possible. However, the online platforms are explicitly recommended for ADR reporting as all relevant information is specifically queried there.

Until 22 November 2017 [29] marketing authorization holders forwarded the ADR reports to the aforementioned competent authorities. After the changes to the pharmaceutical legislation in 2012 marketing authorization holders had to report transitionally to BfArM or PEI, and additionally to the European Medicines Agency (EMA). However, this transitional period ended on 22 November 2017 and BfArM’s ADR database was closed. From that date onwards marketing authorization holders, BfArM, and PEI now forward serious and non-serious ADRs directly to the EMA.

The public access to the restricted set of data elements of BfArM’s ADR database is no longer available since the closure of the database [29]. Due to data privacy requirements, it is not possible to make the individual case reports available to the readership. Nevertheless, researchers and/or readers who are interested can perform the same analysis in the ADR database EudraVigilance of the EMA [30]. However, different levels of access are granted for different stakeholders [31].

### BfArM’s ADR database

BfArM’s ADR database contains about 555,000 ADR reports from Germany up to the data lock point November 22, 2017. The majority of these ADR reports (69.8%) were reported spontaneously (voluntary reporting), whereas 28.2% were reported in studies. In 2.0% it was unknown whether the ADR report originated from spontaneous reporting or from a study [25]. We restricted the present analysis to spontaneous reports for consistency and to avoid any bias through stimulated reporting. In the vast majority of these spontaneous reports a HCP (82.5%) was involved in the reporting of the ADR. In contrast, in 15.6% of the spontaneous reports a non-HCP reported (in 4.5% both, a HCP and a non-HCP reported, and in 6.4% the reporter was unknown).

In the database, drugs are coded according to the WHO Drug Dictionary [32] and the Anatomical Therapeutic Chemical (ATC) classification system [33]. ADRs are coded using the Medical Dictionary for Regulatory Activities (MedDRA) terminology [34]. Both terminologies include five different hierarchical levels for coding and, thus for the analysis of the reported drug substances and ADRs, respectively. The five hierarchical levels represent different levels of analysis with regard to granularity and specificity. In both the highest level of the terminology represents the analysis level of aggregated data (coarse-grained data) with lowest specificity. In contrast, the lowest level of the terminology represents the finer-grained analysis level with highest specificity.

According to the legal definition an ADR is a noxious and unintended reaction caused by a medicinal product [35]. In 2012 the definition of an ADR was extended to the use outside the marketing authorisation including off-label use, overdose, misuse, abuse, and medication errors [36]. A more detailed description of the changes to the legal reporting obligation in the time period from 1987 to 2016 is published elsewhere [25]. The defined time period of our analysis covers both, the new and the old legal definition. For consistency, we restricted our analysis to ADRs associated with the intended use of a drug.

### Identification of cases and reference group

**Fig. 1:**
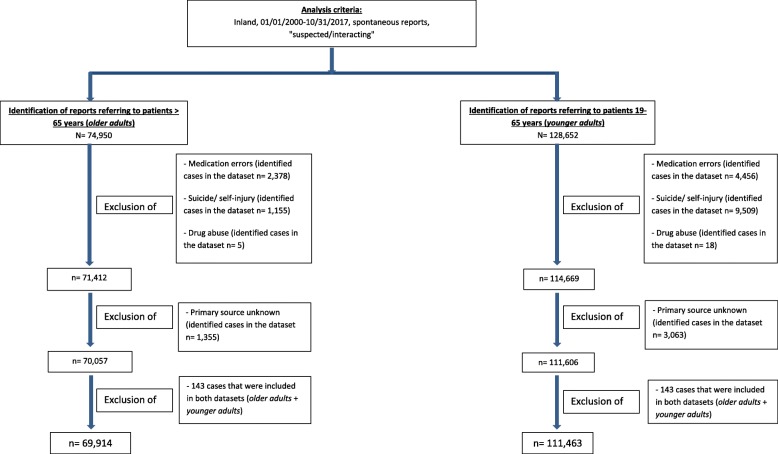
Flowchart: identification of ADR reports for *older adults* and *younger adults*

We identified all spontaneous reports of ADRs referring to patients > 65 years (“*older adults*” aligned with the most frequently applied definition for older adult in developed countries [37]), registered between 01/01/2000–10/31/2017, from Germany (*n* = 74,950) in which drugs were designated as “suspected/interacting” (Fig. 1). All ADR reports coded as medication errors, intentional suicide/self-injury, or drug abuse were excluded by application of respective standardised MedDRA queries [25, 34] (*n* = 71,412). Subsequently, 1355 cases with an unknown primary source were excluded (resulting in *n* = 70,057). In order to analyse i) if more ADR reports of *older adults* are contained in BfArM’s ADR database, and ii) if some of the reported characteristics are more often reported in ADR reports of *older adults* a reference group with patients aged 19–65 years (“*younger adults*”) was generated. For this reference group the same inclusion and exclusion criteria were used (*n* = 111,606). We excluded 143 cases contained in both datasets. Finally, the dataset *older adults* consisted of 69,914 reports whereas the dataset of *younger adults* included 111,463 reports.

### Assessment of ADR reports with regard to quality of documentation and causal association

Due to the large sample size in our analysis (*n* = 69,914 reports) it was not possible to assess each case individually. Instead, we assessed a random sample of 250 ADR reports of *older adults*. This random sample was drawn by using the sample function in R [38]. First, 15 of the randomly selected cases were assessed together by the three evaluators KJ (physician), BS (physician), and DD (pharmacist) in order to harmonise the application of the VigiGrade completeness score [39] and the WHO criteria [40]. VigiGrade evaluates the documentation quality of the ADR reports. A report with a completeness score higher than 0.8 is considered as well documented [39]. The WHO criteria were applied to assess the causal relationship between administration of the suspected drug substances and the ADR. After 50 cases had been assessed we calculated the mean completeness score and its standard deviation (SD). Based on this result we estimated how many cases we would have to evaluate to achieve a completeness score of 0.8. According to this calculation a random sample of 250 cases was necessary. Therefore, we set the case number to 250 for our assessment of quality of documentation and causal association.

The calculation of the completeness score (VigiGrade, [39]) was, however, modified as it was not computed for every reported drug-ADR pair (in case more than one ADR had been reported) and then aggregated to an average, to yield an overall score for the corresponding report. Instead, the score was only calculated for the leading ADR [41].

Finally, the completeness score of our 250 randomly selected cases was 0.75 (95% CI = [0.69–0.81]) with the upper limit of the confidence interval including 0.8. “Time to onset” was the most imprecise criterion (40.4% of reports) due to the fact that it was not documented exactly (19.2%) or was even missing (21.2%).

The assessment of the causal relationship based on the WHO criteria [40] was chosen since it is an internationally used method and due to already existing experiences of the study team regarding its application. In 199/250 reports (79.6%) the causal relationship was considered to be “at least possible” (i.e. 1.6% (4/250) certain plus 22.0% (55/250) probable plus 56.0% possible (140/250)). Hence, if the random sample was representative for the whole dataset, one could expect a dataset of well-documented cases in which about 80% of the reported ADRs have an “at least possible” causal relationship.

### Strategy of analysis

For each group we analysed the number of reports per year, demographic parameters, reported history, seriousness criteria, administration route of the applied drugs, the drugs most frequently reported as suspected together with their most frequently reported ADRs, and the 20 ADRs which were reported most often (irrespective of the drug concerned). Additionally, age-stratified analyses (age intervals: 66–75, 76–85, 86+) were performed in *older adults*.

In order to analyse the reported history, suitable hierarchy levels of the MedDRA terminology [34] were selected. According to the legal definition, an ADR was considered serious if it led to death, was life-threatening, required or prolonged hospitalisation, resulted in persistent or significant disabilities, and/or was a congenital anomaly/birth defect [42]. Hence, this classification of seriousness of the ADR report may differ from the clinical severity of the perceived ADR.

For an overview on drugs classes frequently suspected to cause an ADR, we performed the analysis on the second level of the ATC-code [32, 33] which is a more aggregated level (with lower specificity). Additionally, the drug substance level was selected for a more specific analysis. The ADRs reported most frequently overall and the ADRs associated with the most frequently reported drug classes and drug substances were analysed in both, *older* and *younger adults* on the preferred term (PT) level of the MedDRA terminology [34].

With regard to PIMS we analysed the number of respective ADR reports separately for *older adults*. For this purpose the PRISCUS list [18] was applied as it was the recommendation used presumably most often by physicians in Germany with regard to drug prescribing in older adults. However, the PRISCUS list was lastly revised in 2011. Hence, we also discuss (see discussion) the 10 drug classes and drug substances most frequently reported as suspected in *older adults* with regard to the recommendations of the Beers Criteria [19].

In general, in *older adults* 88,968 suspected drug substances and 206,666 ADRs (PT-level) were coded compared to 136,791 suspected drug substances and 338,046 ADRs (PT-level) in *younger adults*. Only 3.2% and 1.7% of the ADR reports for the *older adults* and *younger adults* were explicitly designated as “interacting”. Hence, these ADR reports were not separately analysed in the context of this study.

The study was designed as a retrospective ADR database analysis which was linked to population-related data about inhabitants [23], assumed drug-exposed inhabitants [4], and DDD per insured person [24], and which incorporates a comparative analysis of ADR reports of *older adults* and *younger adults*.

### Number of DDD per insured person

In order to describe the prescribing behaviours in Germany with rising age we extracted the number of defined daily doses (DDD) per insured person per age group for each of the years 2000–2016 in the German drug prescription reports [24]. Averages (+/−SD) of the mean number of DDD per insured person were calculated for the 16 years per age group. The average number of DDD per insured person of the 16 years per age group was divided by 365 days to calculate the mean number of DDD used per day per insured person per age group.

The drug prescription reports contain all outpatient drug prescriptions of statutory insured patients [24]. Hence, the drug prescription report covers about 80–90% of the German population. The number of prescribed drugs is not patient-related and is available in DDD only. Further limitations refer to missing data on privately insured patients, over-the-counter (OTC) drug use, and inpatient treatments. There is also no exact data referring to the DDD per insured males/females.

### Number of inhabitants and assumed number of drug-exposed inhabitants

The exact number of drug-exposed inhabitants and drug-exposed males/females in Germany is unknown as already described in the previous section [24]. Hence, data about the German population distributed by age and gender for each of the years 2000–2016 (since data of 2017 were limited to October) was extracted from the GENESIS database of the Federal Statistical Office [23] to calculate reporting rates. First, averages (+/−SD) were calculated for the number of ADR reports divided by the number of inhabitants identified for the 16 years for i) each age group, and ii) each of the reported seriousness criteria in the age and gender-stratified analysis. The results are presented as the number of ADR reports per 100,000 inhabitants. However, not all inhabitants are exposed to medication and the proportion of drug exposure may vary between age and gender. Therefore, we estimated the number of assumed drug-exposed inhabitants and drug-exposed males/females based on the number of German inhabitants and German males/females per age group for each year multiplied by the proportion of drug-exposed patients published by a study about the medication use of German adults (DEGS1) [4]. In order to match the conditions of that study, the analysis was adapted to the period of the aforementioned study (2008–2011). Averages (+/−SD) were calculated for the number of ADR reports divided by the number of assumed drug-exposed inhabitants identified for each age group for each of the 4 years. The results are presented as the number of ADR reports per 100,000 assumed drug-exposed inhabitants. Both calculations were based on the date of the ADR report and not of the ADR. However, any inaccuracy would apply to all years, thus diminishing any effects.

### Statistical analysis

Means and medians were calculated for the patients’ age, the annual increase of ADR reports, and frequency distributions for all other results. The chi-squared test was applied to assess differences between the frequency distributions of the datasets for *older adults* and *younger adults*. *P*-values below 0.05 were considered statistically significant. Odds ratios with Bonferroni adjusted confidence intervals (CI) to account for multiple testing were calculated for demographic parameters, comorbidities, the drug classes and drug substances reported most often and their respective ADRs reported most frequently, and for the 20 ADRs reported most frequently, irrespective of the drug concerned.

To analyse if the number of reports for *older adults* have increased proportionally to the number of reports for *younger adults* a ratio (*older adults*/*younger adults*) was calculated for each year.

Regression slopes for the number of ADR reports per 100,000 *older adults* and *younger adults* per year were estimated using linear regression analysis. In order to model the differences in the yearly increase of the slopes for ADR reports per 100,000 *older adults* vs. *younger adults,* an interaction effect between the number of ADR reports per 100,000 *younger adults* and years was included. Differences in the variances of the two groups were taken into account by weighting the observations in the linear model by inverse residuals.

Wilcoxon-Mann-Whitney test was used to detect differences in the medians of the number of ADR reports per 100,000 German males/females for each age group.

All analyses were performed using R, version 3.3.3. The study was approved by the local ethics committee of the Medical Faculty of Bonn (009/17).

## Results

### Characteristics of the reports

Overall age groups more ADR reports referred to females than to males (absolute numbers, without any relation to inhabitants and drug-exposed inhabitants) (Table 1). The relative proportion was slightly higher in *younger adults* than in *older adults* (60.3% vs. 55.9%, OR 0.8 [0.8–0.9]), and increased with rising age within *older adults*.

**Table 1:** Demographic parameters, comorbidities and reported seriousness criteria in *younger adults*, *older adults* and stratified age groups

	*Younger adults* (19–65) (*n* = 111,463)	*Older adults* (> 65) (*n* = 69,914)	OR *older adults* (> 65) vs. *younger adults* (19–65) [+/− adj. CI]	Patients aged 66–75 (*n* = 37,370)	OR patients aged 66–75 vs. *younger adults* [+/− adj. CI]	Patients aged 76–85 (*n* = 24,149)	OR patients aged 76–85 vs. *younger adults* [+/− adj. CI]	Patients aged ≥86 (*n* = 5649)	OR patients aged ≥86 vs. *younger adults* [+/− adj. CI]
Demographic parameters									
mean age (+/− SD) [years]^b^	46.4 (+/− 12.8)	75.4 (+/−7.2)	–	70.5 (+/− 2.8)	–	79.7 (+/− 2.7)	–	89.1 (+/− 2.9)	–
median, [IQR] [years]^b^	48 [37–57]	75 [70–80]	–	70 [68–73]	–	79 [77–82]	–	88 [87–91]	–
female	60.3% (67,249)	55.9% (39,065)	0.8 [0.8–0.9]^a^	52.8% (19,731)	0.7 [0.7–0.8]^a^	58.2% (14,049)	0.9 [0.9–0.9]^a^	68.3% (3861)	1.4 [1.3–1.5]^a^
male	38.4% (42,824)	43.2% (30,230)		46.4% (17,355)		41.0% (9907)		30.9% (1744)	
unknown	1.2% (1390)	0.9% (619)		0.8% (284)		0.8% (193)		0.8% (44)	
Reported patients’ history									
hypertension^c^	9.2% (10,302)	24.5% (17,105)	3.2 [3.1–3.3]^a^	22.8% (8538)	2.9 [2.8–3.1]^a^	27.5% (6652)	3.7 [3.5–3.9]^a^	28.0% (1583)	3.8 [3.5–4.2]^a^
cardiac disorders^d^	7.3% (8180)	24.5% (17,163)	4.1 [3.9–4.3]^a^	20.8% (7776)	3.3 [3.2–3.5]^a^	29.5% (7115)	5.3 [5.0–5.6]^a^	33.6% (1898)	6.4 [5.8–7.0]^a^
diabetes ^e^	4.3% (4830)	11.2% (7837)	2.8 [2.6–3.0]^a^	10.8% (4047)	2.7 [2.5–2.9]^a^	12.5% (3012)	3.2 [2.9–3.4]^a^	11.4% (643)	2.8 [2.5–3.3]^a^
renal disorders^f^	2.8% (3138)	8.9% (6224)	3.4 [3.2–3.6]^a^	7.1% (2670)	2.7 [2.4–2.9]^a^	11.0% (2646)	4.3 [3.9–4.6]^a^	13.4% (759)	5.4 [4.7–6.1]^a^
hepatic impairments^g^	3.3% (3669)	2.5% (1765)	0.8 [0.7–0.8]^a^	2.9% (1068)	0.9 [0.8–1.0]	2.4% (569)	0.7 [0.6–0.8]^a^	1.6% (90)	0.5 [0.3–0.7]^a^
Reported seriousness criteria^h^									
serious	78.9% (87,954)	83.9% (58,681)	1.4 [1.3–1.5]^a^	82.1% (30,669)	1.2 [1.2–1.3]^a^	84.8% (20,482)	1.5 [1.4–1.6]^a^	88.2% (4982)	2.0 [1.8–2.3]^a^
death	3.4% (3755)	9.1% (6340)	2.9 [2.7–3.0]^a^	6.9% (2595)	2.1 [2.0–2.3]^a^	10.6% (2570)	3.4 [3.2–3.7]^a^	15.7% (886)	5.3 [4.7–6.0]^a^
hospitalization	32.7% (36,460)	40.2% (28,094)	1.4 [1.3–1.4]^a^	37.8% (14,131)	1.3 [1.2–1.3]^a^	43.4% (10,490)	1.6 [1.5–1.7]^a^	46.1% (2603)	1.8 [1.6–1.9]^a^
life-threatening	8.2% (9171)	11.9% (8332)	1.5 [1.4–1.6]^a^	11.3% (4223)	1.4 [1.3–1.5]^a^	13.1% (3172)	1.7 [1.6–1.8]^a^	14.6% (825)	1.9 [1.7–2.1]^a^
disabling	2.7% (3020)	3.0% (2118)	1.1 [1.0–1.2]	3.2% (1179)	1.2 [1.1–1.3]^a^	3.0% (731)	1.1 [1.0–1.3]	2.7% (151)	1.0 [0.8–1.3]

^a^: OR = 1 is not included; OR > 1 reported more often in *older adults* or the stratified age groups; OR < 1 reported more often in *younger adults*

^b^ in some cases only the age group (e.g. 7. decade; *older adults* (> 65)) and not the exact age of the patient was reported. If so, these patients were not included in the calculation of the average and median age for *older adults*, *younger adults*, and stratified age groups

^c) -g)^ suitable hierarchical levels of the MedDRA terminology were chosen for the analysis of the reported patients’ history [25]. c) High Level Group Term vascular hypertensive disorder; d) System organ class cardiac disorders; e) High level term diabetes mellitus including subtypes; f) High Level Group Term renal disorders exclusive nephropathies; g) High Level Group Term hepatic and hepatobiliary disorders

^h^ according to the legal definition an ADR was considered serious if it led to death, was life-threatening, required or prolonged hospitalisation, resulted in persistent or significant disabilities, and/or was a congenital anomaly/birth defect [42]

Table 1 shows the absolute numbers of ADR reports and the calculated odds ratios with Bonferroni adjusted confidence intervals for the demographic parameters, the reported comorbidities and the reported seriousness criteria of the patients. The dataset *younger adults* served as a reference for the calculation of the odds ratios. One ADR report may inform about more than one comorbidity and seriousness criteria. Hence, the number of reported comorbidities and seriousness criteria may exceed the number of ADR reports

The reports of *older adults* were more often designated as “serious” (83.9% vs. 78.9%; *p* < 0.001) or “required or prolonged hospitalisation” (40.2% vs. 32.7%; < 0.001), and were even 3 times more often designated as “fatal” (9.1% vs. 3.4%; < 0.001) compared to the reports of *younger adults*.

More comorbidities were reported in *older adults* compared to *younger adults*. For instance, pre-existing vascular hypertensive disorders and renal disorders were mentioned in 24.5 and 8.9% of the reports from *older adults* compared to 9.2 and 2.8% of the reports from *younger adults* (OR 3.2 [3.1–3.3], OR 3.4 [3.2–3.6]) (Table 1). There were no substantial differences regarding either the oral or intravenous route of administration between *older adults* and *younger adults*.

### Annual number of ADR reports (absolute numbers)

The number of ADR reports contained in the ADR database (absolute numbers, without any relation to inhabitants and assumed drug-exposed inhabitants) increased from 2000 to 2016 for *younger adults* and *older adults* with an annual mean increase of 177 and 165 ADR reports, respectively. The calculated ratio of ADR reports for *older adults*/*younger adults* slightly increased from 0.4 in the year 2000 to 0.7 in the year 2017 (mean ratio for the time period 2000–2017: 0.6; range: 0.4–0.8). The age-stratified mean increase of the number of ADR reports per year for the age groups 66–75 years and 76–85 years was approximately the same (both 66 reports/year), while it was notably lower for the age group 86+ years (15 reports/year) (see Supplementary Figure 1 and Supplementary Table 1, Additional file 1).

### Number of reports in relation to inhabitants, assumed drug-exposed inhabitants, and DDD per insured person

**Fig. 2:**
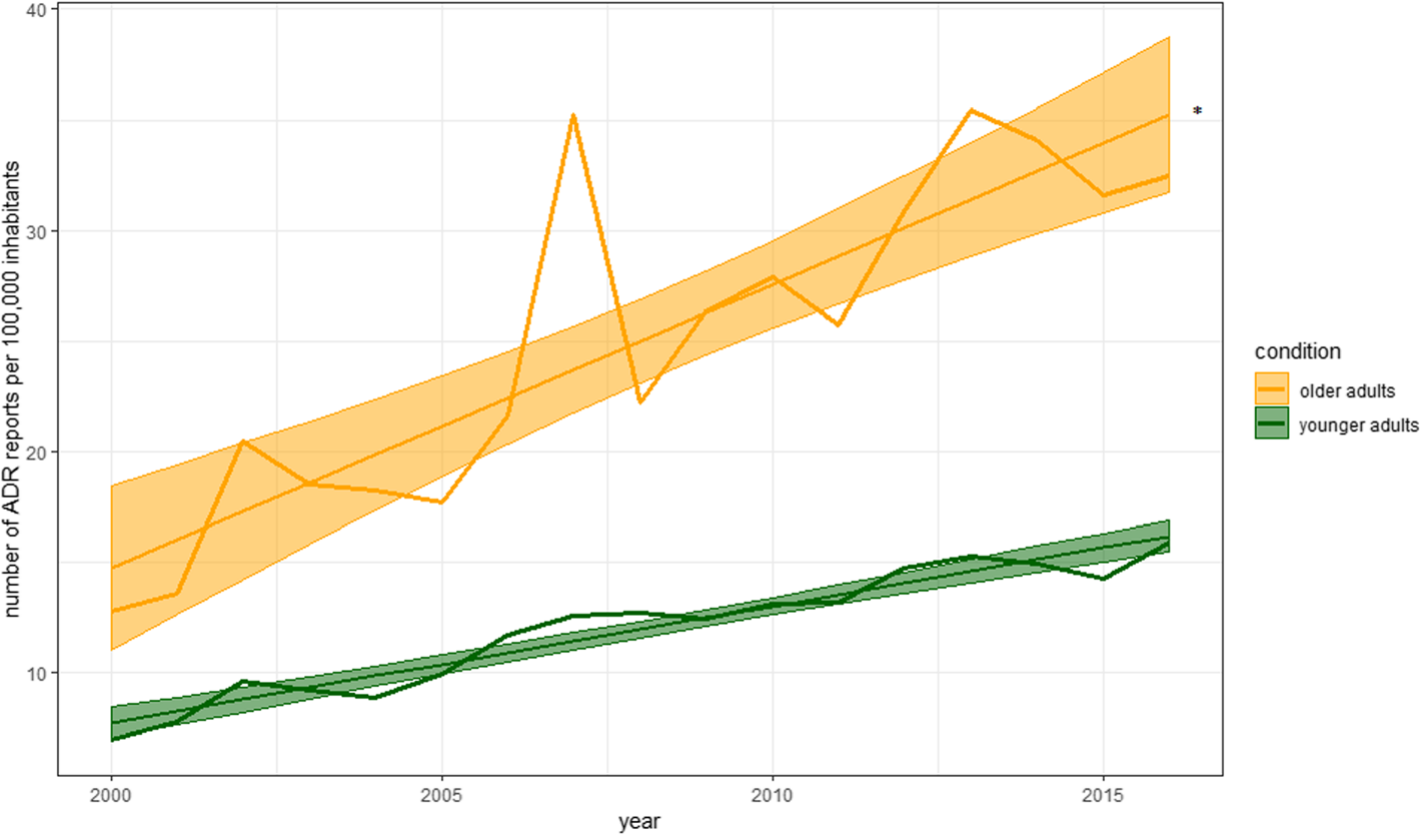
Number of ADR reports per 100,000 younger/older German inhabitants per year. *interaction test of the slopes: *p* < 0.001; slope older adults: 1.3 [0.9-1.7]; slope younger adults: 0.5 [0.5-0.6]. Figure 2 shows the number of ADR reports for *younger adults* per 100,000 German inhabitants (19–65) and the number of ADR reports for *older adults* per 100,000 German inhabitants (> 65) [23] per year. The increases in the number of ADR reports for *older adults* and *younger adults* are presented as weighted linear regression slopes. There was a significant higher increase of the slope for the number of reports per 100,000 older adults than per 100,000 younger adults (*p* < 0.001). The obvious higher number of ADR reports for *older adults* in 2007 is mainly due to reports for rofecoxib (withdrawn in 2004). Roughly 30.0% of these ADR reports in 2007 referred to rofecoxib as suspected drug substance compared to 5.2% of the reports for *younger adults*. About 98.7% of the reports concerning rofecoxib in 2007 were reported by lawyers. Hence, the delayed increase of the number of ADR reports referring to rofecoxib may likely be due to lawsuit after its withdrawal. The limitations of both data sources have to be considered [23, 25]

**Fig. 3:**
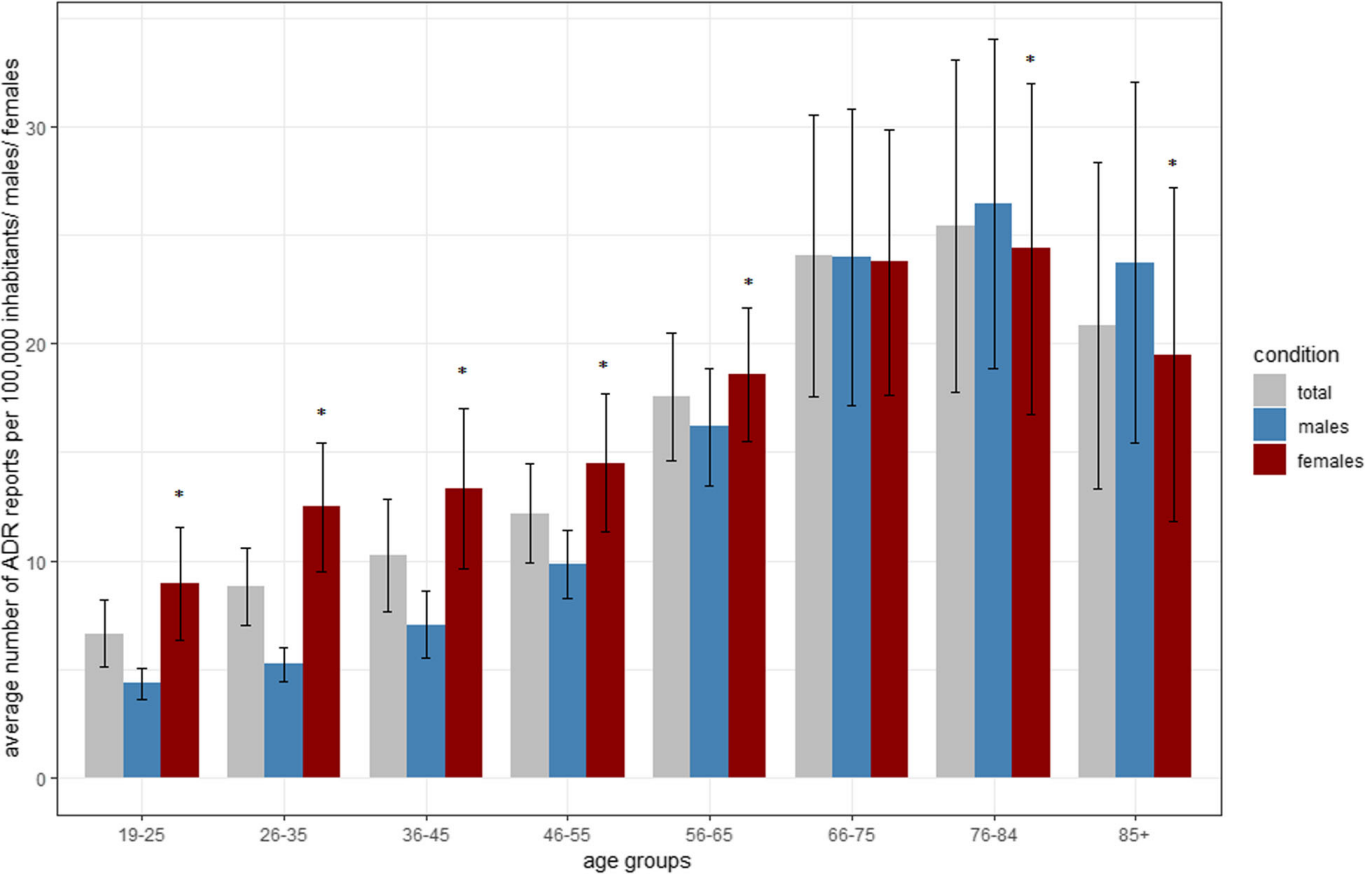
Average number of ADR reports per 100,000 German inhabitants distributed by age and gender. *Wilcoxon-Mann-Whitney test < 0.05. The Fig. 3 shows the average number (+/− SD) of ADR reports per 100,000 German inhabitants distributed by age and gender [23]. The age groups were adapted for this analysis since inhabitants older than 85 years could not be stratified further in the database queried. All ADR reports (male, female and unknown gender) were considered for the calculation of the total average number of spontaneous reports per 100,000 inhabitants (grey bars). Thus, the grey bars possibly do not lie exactly in the middle between the blue and red bars for males and females

**Fig. 4:**
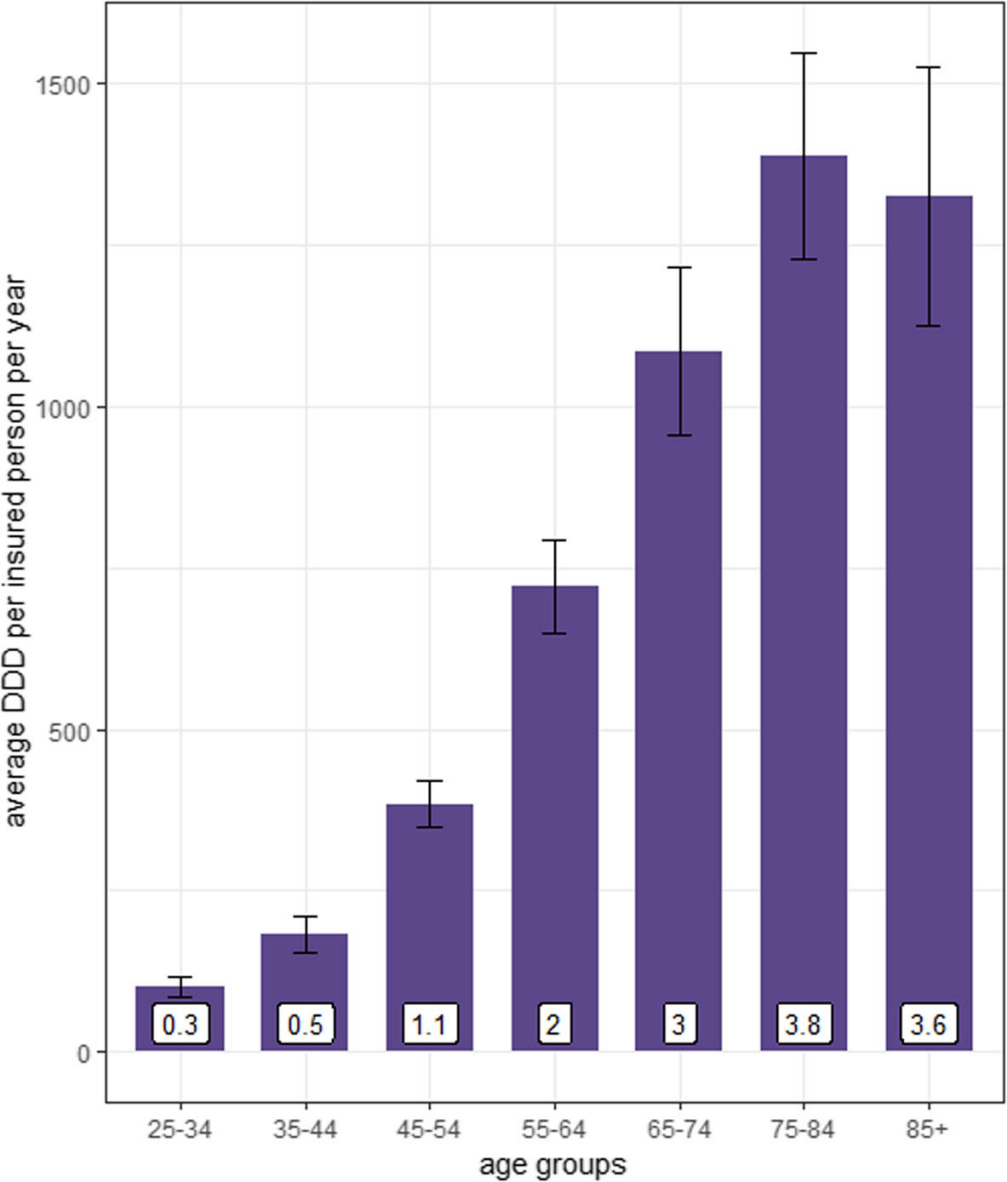
Average number of DDD per insured person. Figure 4 shows the average (+/− SD) of DDD per insured person per age group per year [24]. The mean DDD per day was inserted at the bottom of the bars for each age group. The data stemmed from the German drug prescription reports for the years 2001–2017. The defined age groups of the drug prescription reports were adapted for this analysis since they did not match the defined age groups of the ADR database analysis. Defined daily dose (DDD): The DDD is based on the amount of active substances or medicinal product that should typically be used for the main indication per day. The DDD does not necessarily reflect the recommended or actual administered dose of a drug substance or medicinal product. It mainly provides a technical means of measurement and comparison [24]

The annual number of ADR reports for *older adults* and *younger adults* per 100,000 inhabitants increased from 2000 (12.7 and 6.9) to 2016 (32.6 and 15.8) (Fig. 2). Analysis of the regression slopes revealed a significantly larger increase in *older adults* (*p*-value for interaction effect < 0.001). Across eight age groups the average number of ADR reports/100,000 inhabitants was highest for the age groups 66–75, 76–84, and 85+ (Fig. 3). This finding remained stable if the number of reports was related to the assumed proportion of drug-exposed inhabitants in the respective age group (see Supplementary Document 1, Additional file 2). Notably, the average number of DDD per insured person per age group increased from the youngest age group (25–34) to the age group 75–84 (Fig. 4). The youngest age group (25–34) used on average 0.3 DDD per insured person per day in contrast to 3.8 DDD per insured person for the age group 75–84.

If the number of ADR reports was set in context to inhabitants and exposure more reports referred to males for the age groups > 65 years per 100,000 inhabitants and for the age group > 70 years per 100,000 drug-exposed inhabitants (see Fig. 3 and Supplementary Document 1 Additional file 2). In relation to the number of inhabitants, slightly more ADR reports for all of the reported seriousness criteria were observed for males (Table 2).

**Table 2:** Reported seriousness criteria per 100,000 inhabitants in the stratified age groups

	Patients aged 66–75 years (*n* = 37,370)	Patients aged 76–84 years (*n* = 22,761)	Patients aged ≥85 years (*n* = 7036)
ADR reports per 100,000 inhabitants			
female	23.7 (+/− 6.1)	24.4 (+/− 7.6)	19.5 (+/− 7.7)
males	24.0 (+/− 6.8)	26.4 (+/− 7.6)	23.7 (+/− 8.3)
ADR reports “serious” per 100,000 inhabitants			
female	19.2 (+/− 6.2)	20.6 (+/− 8.0)	17.2 (+/− 8.0)
male	20.1 (+/− 6.5)	22.8 (+/− 7.7)	21.0 (+/− 8.1)
ADR reports “death” per 100,000 inhabitants			
female	1.4 (+/− 0.6)	2.4 (+/− 1.6)	3.0 (+/− 3.2)
male	2.0 (+/− 0.6)	3.0 (+/− 1.2)	3.5 (+/− 2.4)
ADR reports “hospitalisation” per 100,000 inhabitants			
female	8.8 (+/− 2.6)	10.9 (+/− 4.9)	9.6 (+/− 5.2)
male	9.6 (+/− 2.6)	11.7 (+/− 4.0)	10.9 (+/− 3.9)
ADR reports “life-threatening” per 100,000 inhabitants			
female	2.5 (+/− 0.8)	3.3 (+/− 1.5)	3.1 (+/− 2.2)
male	3.2 (+/− 0.9)	3.7 (+/− 1.3)	3.5 (+/− 1.8)

Table 2 shows the average number (+/− SD) of ADR reports per 100,000 German inhabitants distributed by gender and reported seriousness criteria. The age groups were adapted for this analysis since inhabitants older than 85 years could not be stratified further in the database queried [23]. One ADR report may inform about more than one seriousness criteria. Hence, one ADR reports can be assigned to several seriousness criteria

### Most frequently suspected drug classes and drug substances

The analysis of the drug classes reported most often as suspected (second level ATC-code) (Table 3) yielded that antithrombotics were reported almost 5 times more often in *older adults* compared to *younger adults* (1st rank; 19.8% of *older adults*; OR 4.6 [4.3–4.9]). Likewise, among the ten drug substances most often suspected in *older adults*, there were six antithrombotics (acetylsalicylic acid was mostly used as an anti-platelet agent, Table 4). Three of the ten drug classes (Table 3) are used for the treatment of nervous system disorders (6th rank psychoanaleptics, 7th rank psycholeptics, and 10th rank analgesics). Antineoplastic agents ranked 2nd, and antiphlogistics and antirheumatics ranked 3rd.

**Table 3:** The ten drug classes (with their drug substances and ADRs) most frequently suspected in *older adults* and *younger adults*

**Rank**	***older adults*(> 65) % most frequently reported drug classes (number of reports) [(%) three most frequently reported suspected drug substances within the respective drug class]**	**OR with Bonferroni adjusted CI (*older adults*vs.*younger adults*)**	**% three most frequently reported ADRs (number of reports) within the respective drug class**	**OR of reported ADRs with Bonferroni adjusted CI (*older adults*vs.*younger adults*)**
1.	19.8% (13,831) antithrombotic agents (B01) [32.0% rivaroxaban, 12.7% phenprocoumon, 11.8% acetylsalicyclic acid]	4.6 [4.3–4.9]^a^	7.6% (1051) gastrointestinal haemorrhage 5.9% (812) cerebral haemorrhage 4.9% (677) haemorrhage	2.3 [1.7–2.9]^a^ 2.3 [1.7–3.1]^a^ 1.3 [1.0–1.7]
2.	9.1% (6336) antineoplastic agents (L01) [7.4% paclitaxel, 6.1% oxaliplatin, 5.6% imatinib]	1.3 [1.2–1.3]^a^	7.3% (463) dyspnea 6.8% (428) diarrhoea 5.9% (375) nausea	1.0 [0.8–1.2] 1.4 [1.1–1.8]^a^ 1.2 [0.9–1.5]
3.	6.9% (4831) antiphlogistics and antirheumatics (M01) [46.6% rofecoxib, 17.1% diclofenac, 9.3% ibuprofen]	1.7 [1.6–1.8]^a^	16.5% (797) hypertension 15.5% (748) cerebral infarction 12.2% (588) death	2.9 [2.3–3.6]^a^ 6.3 [4.6–8.7]^a^ 13.3 [8.0–21.9]^a^
4.	6.4% (4454) systemic antibiotics (J01) [15.6 levofloxacin, 13.7% ciprofloxacin, 11.4% moxifloxacin]	0.8 [0.8–0.9]^a^	9.1% (406) diarrhea 5.0% (221) nausea 4.9% (218) pruritus	1.2 [0.9–1.4] 0.7 [0.5–0.9]^a^ 0.6 [0.5–0.8]^a^
5.	6.0% (4225) agents acting on the renin-angiotensin system (C09) [19.5% ramipril, 9.5% enalapril, 7.9% valsartan]	2.2 [2.0–2.4]^a^	8.1% (344) angioedema 8.0% (340) dizziness 5.4% (230) nausea	0.9 [0.6–1.2] 1.1 [0.8–1.4] 1.0 [0.7–1.4]
6.	4.7% (3273) psycholanaleptics (N06) [15.0% mirtazapine, 10.6% venlafaxine, 9.9% rivastigmin]	0.7 [0.7–0.8]^a^	8.5% (279) hyponatraemia 6.7% (218) dizziness 6.6% (217) nausea	6.9 [4.6–10.3]^a^ 1.2 [0.9–1.5] 1.1 [0.9–1.5]
7.	4.5% (3138) psycholeptics (N05) [22.8% risperidone, 11.9% quetiapine, 11.4% olanzapine]	0.4 [0.4–0.5]^a^	6.0% (188) drug interaction 5.1% (161) somnolence 4.0% (125) parkinsonism	1.6 [1.2–2.2]^a^ 2.3 [1.6–3.3]^a^ 1.8 [1.2–2.6]^a^
8.	4.0% (2764) lipid modifying agents (C10) [33.5% simvastatin, 23.9% atorvastatin, 11.9% fluvastatin]	1.2 [1.1–1.3]^a^	22.7% (628) myalgia 13.4% (370) blood creatine phosphokinase increased 12.9% (356) rhabdomyolysis	0.6 [0.5–0.8]^a^ 0.8 [0.6–1.0] 1.9 [1.4–2.5]^a^
9.	3.9% (2747) antidiabetics (A10) [19.5% metformin, 17.0% insulin human, 8.5% glibenclamid]	1.5 [1.4–1.7]^a^	21.5% (590) hypoglycaemia 7.2% (198) lactic acidosis 5.9% (161) nausea	2.4 [1.8–3.0]^a^ 2.8 [1.7–4.3]^a^ 0.9 [0.6–1.3]
10.	3.7% (2581) analgesics (N02) [25.2% metamizole, 14.8% fentanyl, 9.0% tramadol]	1.0 [0.9–1.1]	10.0% (259) nausea 6.9% (177) vomiting 6.2% (161) agranulocytosis	1.0 [0.7–1.3] 1.3 [0.9–1.8] 1.0 [0.7–1.5]
rank	***younger adults*(19–65) % most frequently reported drug classes (number of reports) [(%) three most reported frequently suspected drug substances within the respective drug class]**	**OR with Bonferroni adjusted CI (*older adults*vs.*younger adults*)**	**% three most frequently reported ADRs (number of reports) within the respective drug class**	**OR of reported ADRs with Bonferroni adjusted CI (*older adults*vs.*younger adults*)**
1.	10.0% (11,126) psycholeptics (N05) [16.8% clozapine, 16.7% risperidone, 15.7% olanzapine]	0.4 [0.4–0.5]^a^	6.0% (670) weight increased 3.8% (426) drug interaction 3.6% (398) leukopenia	0.1 [0.1–0.3]^a^ 1.6 [1.2–2.2]^a^ 0.8 [0.5–1.2]
2.	7.5% (8400) systemic antibiotics (J01) [13.1% moxifloxacin, 11.5% clindamycin, 11.4 ciprofloxacin]	0.8 [0.8–0.9]^a^	8.0% (672) rash 7.9% (667) diarrhoea 7.9% (667) pruritus	0.6 [0.4–0.8]^a^ 1.2 [0.9–1.4] 0.6 [0.5–0.8]^a^
3.	7.4% (8225) antineoplastic agents (L01) [11.6% paclitaxel, 6.5% docetaxel, 6.5% oxaliplatin]	1.3 [1.2–1.3]^a^	7.3% (601) dyspnea 5.4% (441) pyrexia 5.1% (416) nausea	1.0 [0.8–1.2] 1.0 [0.8–1.3] 1.2 [0.9–1.5]
4.	6.4% (7188) psychoanaleptics (N06) [15.6% venlafaxine, 12.4% mirtazapine, 9.8% duloxetine]	0.7 [0.7–0.8]^a^	5.9% (423) nausea 5.8% (417) dizziness 4.8% (344) drug interaction	1.1 [0.9–1.5] 1.2 [0.9–1.5] 1.2 [0.9–1.7]
5.	5.1% (5689) immunostimulants (L03) [25.0% interferon, 22.4% glatiramer, 21.9% interferon beta-1a]	0.1 [0.1–0.1]^a^	18.0% (1022) multiple sclerosis relapse 4.7% (266) pyrexia 4.6% (260) dyspnoea	0.1 [0.0–0.3]^a^ 1.8 [1.0–3.4] 0.7 [0.3–1.8]
6.	5.1% (5676) antithrombotic agents (B01) [20.6% rivaroxaban, 13.5% phenprocoumon, 9.9% enoxaparin]	4.6 [4.3–4.9]^a^	6.5% (367) thrombocytopenia 6.3% (358) pulmonary embolism 3.7% (211) haemorrhage	0.7 [0.5–0.8]^a^ 0.4 [0.3–0.5]^a^ 1.3 [1.0–1.7]
7.	4.9% (5515) immunosupressivs (L04) [28.7% etanercept, 15.6% fingolimod, 13.1% ciclosporin]	0.6 [0.5–0.6]^a^	4.4% (243) multiple sclerosis relapse 3.4% (189) diarrhoea 3.4% (186) nausea	0.0 [0.0–0.2]^a^ 0.8 [0.5–1.4] 0.8 [0.5–1.4]
8.	4.8% (5323) sex hormones (G03) [12.9% dienogest/ethyinylestradiol, 11.6% drospirenone/ethinylestradiol, 7.5% ethinylestradiol/levonorgestrel]	0.1 [0.1–0.1]^a^	11.1% (590) pulmonary embolism 8.2% (438) deep vein thrombosis 5.2% (279) unintended pregnancy	0.5 [0.3–1.1] 0.4 [0.2–1.1] -
9.	4.7% (5228) antiepileptics (N03) [16.5% carbamazepine, 15.6% levetiracetam, 15.3% pregabalin]	0.6 [0.5–0.6]^a^	7.5% (392) seizure 5.1% (266) dizziness 4.9% (257) hyponatriaemia	0.6 [0.4–0.9]^a^ 1.7 [1.2–2.4]^a^ 1.3 [0.9–1.8]
10.	4.3% (4740) antiphlogistics and antirheumatics (M01) [22.6% rofecoxib, 19.2% diclofenac, 18.4% ibuprofen]	1.7 [1.6–1.8]^a^	6.5% (306) hypertension 6.1% (287) nausea 5.7% (269) dizziness	2.9 [2.3–3.6]^a^ 0.7 [0.5–1.0] 0.7 [0.5–0.9]^a^

^a^OR = 1 is not included; OR > 1 reported more often in *older adults*; OR < 1 reported more often in *younger adults*

Table 3 shows the relative and absolute numbers of ADR reports for the ten drug classes reported most frequently as suspected in *older adults* (> 65) and *younger adults* (19–65), with their three most frequently suspected *drug substances* in relative numbers, and the three most frequently reported ADRs within the respective drug class in relative and absolute numbers. For the analysis of the drug classes the second level, and for the analysis of the drug substances the fifth level of the ATC-code was applied [24]. For the analysis of ADRs reported most frequently the PT-level of the MedDRA terminology [25] was used. One ADR report can contain several drug substances and classes as suspected (hence, multiple assignment of one report to more than one drug class is possible) and inform about several ADRs. Therefore, the number of drug substances and ADRs exceeds the number of ADR reports. The table presents the most frequently reported ADRs within the respective drug class independent of the applied drug substance. Hence, the three most frequently reported ADRs related to the respective drug class may not necessarily be identical to the three most often reported drug substances of the respective drug class. Different drug substances belonging to the same respective drug class may account for the discrepancies in ADRs between *older adults* and *younger adults*. For example, “thrombocytopenia” as the ADR most often reported in *younger adults* for the drug class antithrombotics was due to heparin administration in 44.9% of the “thrombocytopenia” cases. Likewise, “pulmonary embolism” was due to certoparin administration in 29.6% of the “pulmonary embolism” cases in *younger adults*. However, rivaroxaban accounted for only 3.3% of these “thrombocytopenia” cases and 15.9% of these “pulmonary embolism” cases although it was the drug substance suspected most often for *younger adults* among the drug class of antithrombotics. In *older adults* rivaroxaban was also the most frequently reported drug substance in the drug class of antithrombotics and accounted for 26.9% of all “gastrointestinal haemorrhage” cases, and was the most reported drug substance in “cerebral haemorrhage”, and “haemorrhage” cases

**Table 4:** The ten drug substances (with their ADRs) most frequently reported as suspected in *older adults* and *younger adults*

**Rank**	***older adults*(> 65)** **% most frequently reported drug substances (number of reports)**	**OR with Bonferroni adjusted CI (*older adults*vs.*younger adults*)**	**% three most frequently reported ADRs (number of reports)**	**OR of reported ADRs with Bonferroni adjusted CI (*older adults*vs.*younger adults*)**
1.	6.3% (4425) rivaroxaban	6.4 [5.7–7.0]^a^	7.8% (346) epistaxis	2.2 [1.3–3.9]^a^
			6.9% (307) cerebral haemorrhage	3.6 [1.7–7.3]^a^
			5.8% (257) haemoglobin decreased	1.5 [0.9–2.6]
2.	3.2% (2253) rofecoxib	3.4 [3.1–3.8]^a^	32.8% (739) cerebral infarction	3.6 [2.6–5.2]^a^
			32.0% (721) hypertension	1.7 [1.3–2.3]^a^
			25.3% (571) death	8.5 [4.9–14.9]^a^
3.	2.5% (1763) acetylsalicylic acid	3.9 [3.4–4.4]^a^	18.3% (323) gastrointestinal haemorrhage	1.4 [0.9–2.2]
			12.4% (218) melaena	1.0 [0.7–1.6]
			9.4% (165) gastric ulcer haemorrhage	1.2 [0.7–2.1]
4.	2.5% (1762) phenprocoumon	3.7 [3.3–4.3]^a^	13.3% (235) gastrointestinal haemorrhage	1.9 [1.1–3.2]^a^
			9.0% (158) drug interaction	1.4 [0.8–2.4]
			8.9% (157) prothrombin time prolonged	0.8 [0.5–1.3]
5.	2.3% (1635) apixaban	9.1 [7.5–11.0]^a^	7.6% (125) cerebral haemorrhage	2.3 [0.8–7.2]
			7.3% (120) haemorrhage	2.0 [0.7–5.9]
			6.6% (108) off label use	1.4 [0.5–3.7]
6.	2.0% (1427) dabigatran	10.6 [8.5–13.3]^a^	10.3% (147) gastrointestinal haemorrhage	2.0 [0.7–5.5]
			7.9% (113) cerebrovascular accident	0.7 [0.3–1.5]
			6.9% (99) haemorrhage	1.0 [0.4–2.6]
7.	1.6% (1118) diclofenac	1.6 [1.4–1.8]^a^	10.0% (112) gastrointestinal haemorrhage	3.0 [1.6–5.6]^a^
			6.9% (77) pruritus	0.6 [0.4–1.0]
			6.5% (73) nausea	0.9 [0.5–1.6]
8.	1.5% (1067) zoledronic acid	2.0 [1.8–2.3]^a^	47.8% (510) osteonecrosis of jaw	1.0 [0.8–1.4]
			11.1% (118) osteonecrosis	0.7 [0.4–1.1]
			9.7% (104) tooth extraction	0.7 [0.4–1.1]
9.	1.4% (956) clopidogrel	3.9 [3.2–4.6]^a^	12.0% (115) gastrointestinal haemorrhage	2.1 [1.0–4.7]
			6.5% (62) thrombocytopenia	0.9 [0.4–1.8]
			5.0% (48) anaemia	1.2 [0.5–3.1]
			5.0% (48) melaena	0.9 [0.4–2.1]
10.	1.3% (925) simvastatin	1.6 [1.4–1.9]^a^	19.7% (182) myalgia	0.4 [0.3–0.6]^a^
			18.8% (174) rhabdomyolysis	1.8 [1.2–2.8]^a^
			15.5% (143) blood creatine phosphokinase increased	0.8 [0.5–1.1]
**rank**	***younger adults*(19–65) % most frequently reported drug substances (number of reports)**	**OR with Bonferroni adjusted CI (*older adults*vs.*younger adults*)**	**% three most frequently reported ADRs (number of reports)**	**OR of reported ADRs with Bonferroni adjusted CI (*older adults*vs.*younger adults*)**
1.	2.9% (3232) levonorgestrel	0	14.0% (451) uterine perforation	–
			13.7% (444) device dislocation	–
			12.2% (395) pregnancy with contraceptive device	–
2.	1.7% (1868) clozapine	0.2 [0.1–0.2]^a^	10.9% (204) pyrexia	1.6 [0.8–3.1]
			10.1% (189) leukopenia	1.4 [0.7–2.8]
			8.1% (152) c-reactive protein increased	0.8 [0.3–2.1]
3.	1.7% (1856) risperidone	0.6 [0.5–0.7]^a^	7.0% (129) weight increased	0.2 [0.1–0.5]^a^
			6.6% (122) galactorrhoea	0.0 [0.0–0.6]^a^
			6.0% (111) akathisia	0.3 [0.1–0.8]^a^
4.	1.6% (1749) olanzapin	0.3 [0.3–0.4]^a^	15.6% (273) weight increased	0.1 [0.0–0.4]^a^
			5.3% (93) blood creatine phosphokinase increased	0.7 [0.2–1.8]
			5.0% (87) alanine aminotransferase increased	0.1 [0.0–1.6]
5.	1.4% (1585) etanercept	0.7 [0.6–0.8]^a^	7.4% (118) condition aggravated	0.9 [0.5–1.6]
			6.5% (103) rheumatoide arthritis	1.6 [0.9–2.7]
			4.9% (78) drug ineffective	0.8 [0.4–1.7]
6.	1.3% (1420) interferon	0.1 [0.1–0.1]^a^	20.8% (295) multiple sclerosis relapse	0.1 [0.0–0.9]^a^
			4.4% (63) pyrexia	1.0 [0.2–5.7]
			3.8% (54) headache	0.3 [0.0–8.3]
7.	1.1% (1272) glatiramer	0.02 [0.01–0.04]^a^	23.0% (293) multiple sclerosis relapse	0.3 [0.0–9.1]
			11.2% (142) dyspnea	–
			7.1% (90) injection site necrosis	1.1 [0.0–36.1]
8.	1.1% (1258) quetiapine	0.5 [0.4–0.6]^a^	7.7% (97) drug interaction	1.3 [0.6–2.5]
			7.7% (97) weight increased	0.1 [0.0–0.7]^a^
			6.0% (76) leukopenia	0.5 [0.2–1.4]
9.	1.1% (1243) interferon beta-1a	0.03 [0.02–0.06]^a^	19.4% (241) multiple sclerosis relapse	0.5 [0.1–4.3]
			8.4% (104) influenza like illness	0.4 [0.0–13.5]
			4.8% (60) alanine aminotransferase increased	1.6 [0.1–20.0]
10.	1.1% (1173) rivaroxaban	6.4 [5.7–7.0]^a^	8.7% (102) menorrhagia	0.0 [0.0–0.1]^a^
			5.5% (65) deep vein thrombosis	0.3 [0.2–0.5]^a^
			5.1% (60) pulmonary embolism	0.4 [0.2–0.6]^a^

^a^OR = 1 is not included; OR > 1 reported more often in *older adults*; OR < 1 reported more often in *younger adults*

Table 4 shows the relative and absolute numbers of ADR reports of the ten drug substances most frequently reported as suspected in *older adults* (> 65) and *younger adults* (19–65) with their relative and absolute numbers of the three most frequently reported ADRs. For the drug substances the fifth level of the ATC-code was applied [24]. For the analysis of ADRs reported most frequently the PT-level of the MedDRA terminology [25] was used. One ADR report can contain several drug substances as suspected and inform about several ADRs. Therefore, the number of drug substances and ADRs exceeds the number of ADR reports. Since we did not perform an individual case assessment for all ADR reports (e.g. with regard to the causal association with the drug intake), it cannot be excluded that the most frequently reported ADRs may also stand in a causal relation to other drug substances that were also reported as suspected within the ADR report. However, one may assume that the three most frequently reported ADRs are more likely to be causally related to the listed drug substance since they are reported so often

In contrast, psycholeptics were the drug class most frequently reported in *younger adults* (10.0% of the reports; OR 0.4 [0.4–0.5], Table 3). Likewise, four of the ten drug substances most frequently suspected within the reports for *younger adults* were antipsychotics (only one being an antithrombotic; rivaroxaban ranking 10th) (Table 4).

Only 3611 (4.1% of 88,968) suspected drug substances reported in *older adults* were PIMs according to the PRISCUS list. Olanzapine was the most often reported PIM in *older adults* (45th rank in *older adults* with 0.5% of *older adults* reports) (see Supplementary Table 2, Additional file 3). In contrast, olanzapine ranked fourth in the reports of *younger adults* (Table 4).

### Most frequently reported ADRs

There is broad consistency along with some differences concerning the 20 ADRs reported most frequently in *older adults* and *younger adults* irrespective of the suspected drug substance (see Supplementary Table 3, Additional file 4). In the top ranks of both, mainly unspecific ADRs (“nausea”, “dizziness”, “dyspnoea”, “diarrhoea”, “pruritus”, “vomiting”, “rash”, “headache”) are listed. Interestingly, those mainly unspecific ADRs were less often reported in patients older than 86 years (see Supplementary Table 3, Additional file 4). The highest odds ratios (and thus more frequently reported in *older adults* compared to *younger adults*) were observed for “gastrointestinal haemorrhage” (15th rank; OR 5.1 [4.2–6.1]), “death” (9th rank; OR 3.8 [3.3–4.4]), “fall” (18th rank; OR 3.0 [2.6–3.6]), and “cerebrovascular accident” (19th rank; OR 3.0 [2.6–3.6]). Conversely, for *younger adults* the lowest odds ratios compared to *older adults* (and thus being more reported in *younger adults*) were found for “urticaria” (12th rank; OR 0.5 [0.4–0.5]), “paraesthesia” (19th rank; OR 0.5 [0.4–0.6]), and “hepatic enzyme increased” (18th rank; OR 0.6 [0.5–0.7]). The calculated odds ratios for “death”, “gastrointestinal haemorrhage”, “fall”, “cerebrovascular accident”, “cerebral infarction”, “syncope”, “cerebral haemorrhage”, and “haemoglobin decreased” increased with rising age. It should be noted though, that “death” itself is not an ADR but an outcome coded by MedDRA terminology [25].

### Drug classes reported as suspected most frequently and their ADRs

The ADRs reported most frequently differed for some drug classes between *older adults* and *younger adults*. This becomes obvious with antithrombotics, psychoanaleptics, and psycholeptics (Table 3). For instance, for antithrombotics, “gastrointestinal and cerebral haemorrhage” were the ADRs reported most frequently for *older adults*. In contrast “thrombocytopenia” and “pulmonary embolism” were the ADRs reported most frequently for *younger adults* (possibly suggesting ineffectiveness of the drug). Similarly, “hyponatraemia” was the ADR reported most frequently for psychoanaleptics in *older adults* but ranked only 29th in the respective reports of *younger adults*.

Different drug substances belonging to the same respective drug class (Table 3) may account for the discrepancies in ADRs between *older adults* and *younger adults* (further description see legend Table 3).

### Drug substances reported as suspected most frequently and their ADRs

Likewise, Table 4 shows that for some drug substances the most frequently reported ADRs between *older adults* and *younger adults* differed. The ADRs most frequently reported for rivaroxaban were “epistaxis” (OR 2.2 [1.3–3.9]), and “cerebral haemorrhage” (OR 3.6 [1.7–7.3]) in *older adults* vs. “menorrhagia” (OR 0.0 [0.0–0.1]), and “deep vein thrombosis” (OR 0.3 [0.2–0.5]) in *younger adults*. Further analysis with regard to rivaroxaban revealed that the indications most often reported differed between *older adults* and *younger adults* (see Supplementary Table 5, Additional file 6). Hence, not only the drug substance itself but the difference in the indications (i.e. the underlying diseases) could have affected the ADR profile. Among the other antithrombotic agents (acetylsalicylic acid (3rd rank), phenprocoumon (4th rank), and apixaban (5th rank)) differences concerning the ADRs most frequently reported were less striking (see Supplementary Table 6, Additional file 7). However, “gastrointestinal haemorrhage” (OR 1.9 [1.1–3.2]) related to phenprocoumon, “cerebral haemorrhage” (OR 2.3 [0.8–7.2]) related to apixaban, “gastrointestinal haemorrhage” related to dabigatran (OR 2.0 [0.7–5.5]) and clopidogrel (OR 2.1 [1.0–4.7]), respectively, were reported more often in *older adults* than *younger adults*. Further differences were observed with regard to the ADRs most frequently reported for risperidone and olanzapine. “Falls” were reported about 10 times more often for risperidone and “parkinsonism” was reported about 4 times more often for olanzapine in *older adults* compared to younger adults.

## Discussion

This study is the first retrospective analysis of ADR reports referring to older adults in the national ADR database of the competent authority BfArM in Germany. In order to strengthen the significance of the ADR database analysis, parallel analysis with other external data sources providing complementary data about the number of inhabitants [23], the medication use (prescription-only medicine and OTC) [4], and drug prescriptions [24] were also conducted. Furthermore, the ADR reports of *older adults* were compared to ADR reports of *younger adults* in order to identify differences among both patient populations. We saw a significant higher increase of ADR reports in *older adults* per 100,000 inhabitants vs. *younger adults* per 100,000 inhabitants in the last years, underlining the importance of ADRs in older adults. Interestingly, the ADRs reported the most frequently differed for some drug classes and drug substances between *older* vs. *younger adults*.

An increase of the absolute number of ADR reports with rising age up to the age group 66–70 years was already shown in our previous descriptive analysis of all ADR reports contained in BfArM’s ADR database [25]. In the present study, however, the number of ADR reports was set in relation to the number of inhabitants and assumed drug-exposed inhabitants distributed by age and gender [4, 23]. We found an increase in the number of ADR reports per 100,000 inhabitants and assumed drug-exposed inhabitants with rising age up to the age groups 76–84 years and 70–79 years, respectively. Our finding may reflect the increase of older inhabitants in the same time frame in Germany [23] which may have led to an increase of drug-exposed inhabitants and, thus, more patients with ADRs.

In an analysis of the global ADR database *Vigibase* the highest mean number of ADR reports per million inhabitants for high-income countries has been observed for the age group 65–74 years [6]. The slight shift compared to our age strata may be explained by differences of the underlying data. Our analysis was restricted to Germany only, whereas the analysis in *Vigibase* included several high-income countries.

The rising frequency of ADRs with older age per inhabitants has also been described in ADR database analysis of other countries [21, 43, 44]. A higher proportion of ADRs in inpatients older than 65 years compared to younger inpatients has been reported in two medical record studies performed in German hospitals as well [10, 45]. Various factors may account for this finding, e.g. a higher proportion of multi-morbid persons and a higher proportion of drug-exposed and polymedicated patients, which has been described in two German surveys [3, 4]. Polypharmacy and comorbidities have been assumed to correlate with the seriousness of spontaneously reported ADRs in a study from Italy [21]. This may also explain the increase of serious ADRs with rising age in our analysis (see below).

ADRs itself and ADR related hospital admissions are associated with costs for the Health Care System [46] which are estimated to be even higher for patients older than 65 years [9]. Assuming that the number of ADR reports will further increase in the future, we would expect almost a doubling of ADR reports per 100,000 older inhabitants (78.9 [62.1–95.7] ADR reports) in the year 2050 based on the linear trend displayed in Fig. 2. If so, a further increase of health care costs can be expected in the future. However, this prediction is associated with considerable uncertainty due to the distance of the year 2050 to the analysed time period (2000–2016) and possible unknown variables (e.g. legislative changes) that may occur in the future and could impact on this scenario.

Known risks for ADRs in older patients are age-related changes in pharmacodynamics and pharmacokinetics, e.g. reduced kidney and liver function leading to a higher variability in drug response [5, 47]. Likewise, we also found a higher proportion of patients with one of the queried comorbidities (e.g. cardiac disorders) with rising age, except for hepatobiliary disorders. The higher number of patients with hepatobiliary disorders in *younger adults* compared to *older adults* could be due to a reduced life expectancy of patients with severe - and thus possibly also more often reported - hepatobiliary disorders. Compared to a German survey [3] the proportion of individuals older than 65 years with hypertension was much lower in our analysis (50% vs. 24.5%). This discrepancy could be due to incomplete or missing data in the ADR reports or differences in the recording of diseases inherent to the different study designs.

In the present study an ADR was considered serious if it led to death, and/or hospitalisation or prolonged hospitalisation, and/or congenital anomalies or was life-threatening [42]. A higher proportion of “serious” ADRs and ADRs “leading to/or prolonging hospitalisation” with increasing age has been seen in spontaneously reported ADRs from Italy and Sweden as well [11, 21]. Likewise, in a German cohort study an increase of ADR related hospital admissions has been reported with increasing age [9]. However, differences regarding the study designs have to be considered.

Like the Swedish study which focussed on fatal ADR reports [11] we observed an increase of ADR reports informing about a fatal outcome with rising age, as well. However, it should be noted that we did not specifically assess fatal ADR reports with regard to their causal relationship. Hence, we cannot elucidate the number of cases in which the fatal outcome was due to other causes like underlying comorbidities or natural death.

As also observed in other ADR database analysis [17, 48, 49] we found a higher absolute number of ADR reports referring to older females with rising age. This finding may be explained by (i) sex differences in pharmacokinetics and pharmacodynamics [50], (ii) differences in reporting behaviours (females tend to report ADRs more often than males [48, 51]), (iii) the higher number of female inhabitants in the older German population [23, 52], and (iiii) more older females in the German population taking drugs and having comorbidities compared to older males [3, 4].

Unexpectedly, slightly more ADR reports referred to *older males* than *females* when related to either 100,000 inhabitants or assumed drug-exposed inhabitants in our analysis. With regard to gender related differences concerning ADRs in older adults there is conflicting data in literature [15, 17, 44, 53–55]. Different study designs (e.g. observational studies versus analysis of ADR reports) and different denominators (e.g. drug prescriptions versus inhabitants) may account for these differences. For instance, female gender as a risk factor for ADRs has been reported in a prospective multicentre cohort study involving three German hospitals and one hospital in Jerusalem overall and for females older than 65 years even after adjusting for age, body mass index and the number of prescribed drugs [53]. In a Swedish study the number of ADR reports for females related to the number of drug prescriptions in DDD was similar or only slightly lower in the age groups 75–84 years and ≥ 85 years but significantly higher in the age group 65–74 years compared to males [17]. In an older study from West Germany Hopf et al. [15] found more ADR reports per 1,000,000 million inhabitants for males from the age group 60–69 years onwards. However, this was only observed before adjusting for drug exposure in DDD [15]. Our results that more ADR reports referred to older males for both denominators (inhabitants and drug exposed-inhabitants) are thus in line with the first but not the second finding (different denominators) from Hopf et al. [15].

In some database analyses a higher proportion of “serious” ADR reports and/or ADR reports with fatal outcome were found in older males [11, 17, 49]. In our study, a slightly higher number of ADR reports for all seriousness criteria in all stratified age groups was only observed when related to 100,000 inhabitants (not for all age groups in absolute numbers). In a French analysis, a preponderance of male gender for serious ADRs in relation to inhabitants has been observed for the age group 60–69 years only [54]. Possibly the higher number of ADR reports per 100,000 older male inhabitants in our analysis may be due to serious ADRs which are more often reported by German physicians [56]. However, as a conclusion from our findings, female gender should not be considered as a risk factor for *all* age groups. Especially in older adults more emphasis should be put on the occurrence of ADRs and serious ADRs in older males.

In the last few years the number of drug prescriptions for antithrombotics (especially for rivaroxaban) increased enormously [24] and drug-exposure in terms of DDD increased with rising age [24]. Likewise, in our analysis almost one fifth (19.8%) of all ADR reports of *older adults* reported an antithrombotic agent as “suspected/interacting” drug (and the number of these reports has increased over the last years). However, we cannot elucidate whether antithrombotics actually cause more ADRs or if these are only reported more frequently, due to the huge number of drug prescriptions. Nevertheless, antithrombotics were identified as the top ranking drugs responsible for ADR in older adults in ADR database studies from Italy and France [21, 57] and in medical record studies from Germany and US [10, 58]. In contrast, psycholeptics ranked first in *younger adults* in our analysis accounting for 10.0% of all reports in *younger adults* (4.5% of all reports in *older adults*). This finding is in line with studies showing that ADRs associated with drugs acting on the nervous system were more often reported for *younger adults* [17, 21] vs. *older adults* [59].

Interestingly, for some drug substances and drug classes the ADRs reported most often differed between *older adults* and *younger adults*. This was striking for rivaroxaban. Differences regarding the reported indications for rivaroxaban between *younger* and *older adults* and, thus, a more common chronic use (e.g. atrial fibrillation) in *older adults* may account for this finding. A cohort study has shown that the risk for bleeding, especially gastrointestinal bleeding, inherently increases with rising age [60], it may then be potentiated by antithrombotics. In this respect, higher numbers of ADR reports with regard to gastrointestinal and nervous system haemorrhages associated with direct oral anticoagulants have been seen in patients aged 60 years or older compared to younger patients in a study performed in two large ADR databases from USA and Japan [61]. Haemorrhages were the cause of death reported most often in the Swedish study of fatal ADR reports [11]. Within these reports, antithrombotics were most frequently suspected. Hence, our data in conjunction with the data from literature underline the recommendation to monitor older patients taking antithrombotics.

Likewise to the increase of prescription-only drugs, the use of OTC drugs increases with rising age [4]. Two out of the 10 most frequently reported drug substances in *older adults* are also available as OTC drugs in Germany (acetylsalicylic acid (3rd) and diclofenac (7th)). In our analysis we cannot differentiate, if acetylsalicylic acid or diclofenac had been prescribed or taken as an OTC drug. However, since OTC drugs may also cause ADRs or interact with prescribed therapy [62] the importance of taking a full medical history inclusive OTC drugs and food supplements still remains.

In our study, “parkinsonism” was reported as an ADR for psycholeptic drugs and olanzapine 1.8 times and 4 times more often in *older adults* compared to *younger adults*, respectively. In general, the prevalence of Parkinson disease increases with rising age [63]. However, “parkinsonism” as an example for an ADR may be difficult to distinguish from the onset of the disease itself, the progression of the disease or signs of aging, which illustrates the challenge of ADR recognition in older adults. Hence, in order to avoid prescription cascades new symptoms should be critically examined and their aetiology clarified.

The exact exposure of older adults with PIM in the German population is unknown. In our analysis PIMs according to PRISCUS [18] were not very frequently reported as suspected in *older adults*. One explanation for this observation could be that non-PIM related ADRs are more frequently in our analysis due to the higher number of drug prescriptions for non-PIMs. This may lead to an underrepresentation of ADRs related to PIMs. In a prospective medical record study performed in Germany the prevalence of ADRs associated with a PIM was rather low [45]. Likewise, more ADR reports related to non-PIMs than to PIMs according to the Laroche list have also been reported in a study conducted in a French Pharmacovigilance database [57]. However, differences in PIM lists and PIM prescription behaviours between Germany and France complicate the comparability of this study with our study. In addition, an underreporting of PIMs e.g. due to fear of legal consequences cannot be excluded. This limitation, however, would probably also apply to the French study.

In our analysis, risperidone and mirtazapine were the psycholeptic and psychoanaleptic drug substances reported most frequently in *older adults*. Both are recommended in the PRISCUS list [18] to be prescribed instead of other psycholeptics and psychoanaleptics. Conversely, the international Beers Criteria [19] advises caution when using both drug substances in older adults and recommend a close monitoring of sodium levels when prescribing mirtazapine and psychoanaleptics. In our analysis “hyponatraemia” was infact about 7 times more often reported for the drug class psychoanaleptics in *older adults* than in *younger adults*.

In the Beers Criteria [19] the chronic use of diclofenac is discouraged in older adults due to an increased risk of gastrointestinal (GI) bleeding. In contrast, diclofenac is not reported as inappropriate drug for older adults in the PRISCUS list [18]. In our analysis, “GI haemorrhage” associated with diclofenac (7th rank) was roughly three times more often reported in *older adults* compared to *younger adults*. It should be noted that diclofenac is also available as an OTC drug in Germany. Hence, diclofenac intake will even be higher, and subsequently may impact on the number of ADR reports referring to diclofenac. In summary, our findings with regard to risperidone, mirtazapine, and diclofenac are consistent with the recommendation of the Beers Criteria.

The seven ADRs reported most frequently for *older adults* and *younger adults* are rather unspecific and may be co-reported to the main ADR triggering the report [25]. Among the 20 ADRs reported most often for *older adults*, were “gastrointestinal haemorrhage”, “death”, “fall”, and “cerebrovascular accident” (see Supplementary File 4, Supplementary Table 3)*.* An increase in the frequency of these four ADRs was observed with rising age in our dataset and is also reported in literature [11, 58]. This observation may reflect the increase of serious ADRs with rising age as discussed above.

Falls in general, as well as ADRs which may favour falls like syncope or confusional states (also more often reported with rising ages in our analysis) are associated with a higher mortality, morbidity and immobility [64, 65]. These may lead to more intense need of care in older adults, resulting in an enormous increase of health care costs [64]. Hence, physicians should critically examine the current and intended drugs taken with respect to their potential to favour falls.

The monitoring of drugs used in older adults remains of major importance since data about efficacy and safety in older adults are still underrepresented in initial drug approval documents [66]. Despite its limitations the spontaneous reporting system has proved to be a useful tool to recognize ADRs after marketing approval [25]. Its strengths are based on a large population coverage including real world data as well as vulnerable patient populations (e.g. older adults, comorbid patients), a long-term data collection, and the inclusion of all types of drugs like OTC drugs [25].

One of its major limitations is the unknown amount of underreporting [67], which may depend on the type of ADR and drugs taken, or the recognition of the symptoms as an ADR, especially in older adults [56]. Another limitation is the lack of matching exact exposure data. As a consequence of these both limitations, exact incidences and prevalences cannot be calculated, which also applies to our results. To address this limitation, we set the number of ADR reports in relation to the number of inhabitants and assumed drug-exposed patients. This allows for an estimation of the dimension but should not be misunderstood as exact prevalences and/or incidences.

The distribution of ADR reports originating from physicians, pharmacists and patients was equal in *older* and *younger adults*. Hence, published differences in reporting behaviours among these three reporter types [25, 56, 68, 69] are not assumed to play a role for the detected differences between *younger* and *older adults* in our analysis.

We could not account for any impact of the medical speciality of the reporter since respective data is only rarely available. The chronological age and biological age may differ individually, as well as the degree of frailty, which also could have an impact that cannot be accounted for in our analysis.

Finally, a full case validation with regard to the causal relationship and the quality and completeness of the reports was not possible due to the large sample sizes. However, we would like to point out that all ADR reports have been submitted to BfArM because the reporter assumed an underlying causal association. However, if an equal distribution of cases with poor documentation quality and lack of causal relationship is expected, the same tendency of the results would be observed with a smaller number of cases.

## Conclusion

In summary, our analysis underlines the need to further investigate ADRs in older adults since these reports are expected to significantly increase in the future. Also, more attention should be payed to the occurrence of ADRs in older males. Moreover, physicians should be aware of different ADRs being associated with the same drug depending on age. Our findings may also be helpful for the regular update of PIMs lists. Physicians should continue their caution and monitoring when prescribing antithrombotics to older adults. Finally, HCPs should report ADRs, particularly in older adults, as this gives regulators and researches the possibility to further investigate ADRs in older adults and to develop strategies to prevent them.

## Supplementary information

**Additional file 1 Supplementary Figure 1**. The number of ADR reports per year for younger adults, older adults, patients aged 66-75 years, patients aged 76-85 years, patients aged ≥ 86 years (absolute numbers). **Supplementary Table 1**. The calculated ratio “number of ADR reports for older adults/number of ADR reports for younger adults” per year.

**Additional file 2 Supplementary Document 1**. The average number of ADR reports per 100,000 inhabitants/males/females and estimation of the number of ADR reports per 100,000 assumed drug-exposed inhabitants/males/females per age group.

**Additional file 3 Supplementary Table 2**. The number of ADR reports of the potentially inappropriate medications (PIMs) contained in the PRISCUS list in *older adults* (> 65 years).

**Additional file 4 Supplementary Table 3**. The 20 ADRs reported most frequently in the ADR reports of *younger adults*, *older adults* and stratified age groups.

**Additional file 5 Supplementary Table 4**. The three drug substances most frequently suspected for the three most frequently reported ADRs in the ADR reports of antithrombotic agents of *younger adults* and *older adults*.

**Additional file 6 Supplementary Table 5**. Characteristics, drug indications, and ADRs in the ADR reports of *younger adults* and *older adults* in which rivaroxaban was suspected before and after extension of the indication (01/13/2012).

**Additional file 7 Supplementary Table 6**. The five most frequently reported ADRs of *younger adults* in which phenprocoumon, acytylsalicyclic acid, and apixaban were reported as suspected drug substance.

## Data Availability

The datasets generated and/or analysed during the current study are not publicly available due data privacy requirements. Researchers and/or readers who are interested can perform the same analysis in the ADR database EudraVigilance of the EMA (public access: http://www.adrreports.eu/en/index.html). However, different levels of access are granted for different stakeholders [22].

## Abbreviations

ADR: Adverse drug reaction
ATC classification: Anatomical Therapeutic Chemical classification
AVP: Drug prescription reports (Arzneiverordnungs-Reporte)
BfArM: Federal Institute for Drugs and Medical Devices
CI: Confidence interval
DDD: Defined daily doses
EMA: European Medicines Agency
HCPs: Health Care Professionals
MedDRA: Medical Dictionary for Regulatory Activities
non-HCPs: Non-Health Care Professionals
OR: Odds ratio
OTC drugs: Over-the-counter drugs
PEI: Paul-Ehrlich-Institut
PIMs: Potentially inappropriate medications
PT: Preferred term
