# Supplementary material for: Adverse drug reactions in older adults: a retrospective comparative analysis of spontaneous reports to the German Federal Institute for Drugs and Medical Devices
Source: BMC Pharmacol Toxicol. 2020 Mar 23;21:25. doi: 10.1186/s40360-020-0392-9 (PMC7092423; doi:10.1186/s40360-020-0392-9)
Supplement: Supplementary file 1 — Additional file 1 Supplementary Figure 1. The number of ADR reports per year for younger adults, older adults, patients aged 66-75 years, patients aged 76-85 years, patients aged ≥ 86 years (absolute numbers). Supplementary Table 1. The calculated ratio “number of ADR reports for older adults/number of ADR reports for younger adults” per year. [file 40360_2020_392_MOESM1_ESM.docx]

**Supplementary Figure 1. The number of ADR reports per year for *younger adults*, *older adults*, patients aged 66-75 years, patients aged 76-85 years, patients aged ≥ 86 years (absolute numbers).**

**
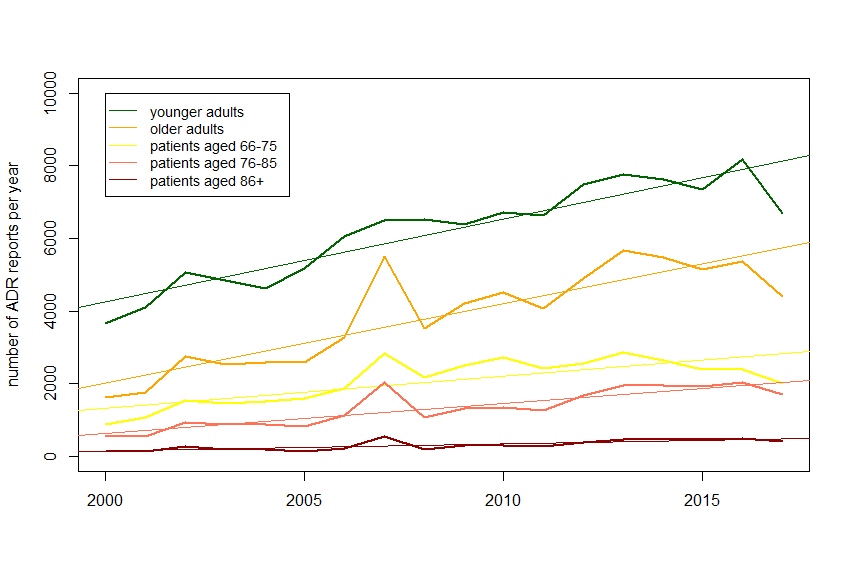
**

Supplementary Figure 1 shows the absolute number of ADR reports for *younger adults* (19-65 years), *older adults* (> 65 years), and the stratified age groups patients aged 66-75 years, patients aged 76-85 years and patients aged ≥ 86 years per year. The stratified age groups are subgroups of the *older adults*. In some cases, only the age group (e.g. 7. decade; older adults (> 65)) and not the exact age of the patient was reported. If so, these patients cannot assigned to the stratified age groups. Hence, the sum of the number of ADR reports of all three stratified age groups is not equal to the number of ADR reports for *older adults*.

The absolute number of ADR reports for *older adults*, *younger adults*, and the stratified age groups increased over the years with an annual mean increase of 165 ADR reports for *older adults*, 177 ADR reports for *younger adults*, 66 ADR reports for patients aged 66-75 and patients aged 76-85, and 15 ADR reports for patients aged ≥ 86, respectively. The obvious higher number of ADR reports for *older adults* (and the stratified age groups) in 2007 is mainly due to reports for rofecoxib (withdrawn in 2004). Roughly 30.0 % of these ADR reports in 2007 contained rofecoxib as suspected drug substance compared to 5.2 % of the reports for *younger adults*.

**Supplementary Table 1. The calculated ratio "number of ADR reports for *older adults*/ number of ADR reports for *younger adults*" per year.**

| year | 2000 | 2001 | 2002 | 2003 | 2004 | 2005 | 2006 | 2007 | 2008 | 2009 | 2010 | 2011 | 2012 | 2013 | 2014 | 2015 | 2016 | 2017 |
| --- | --- | --- | --- | --- | --- | --- | --- | --- | --- | --- | --- | --- | --- | --- | --- | --- | --- | --- |
| ratio *older adults*/ *younger adults* | 0.44 | 0.43 | 0.54 | 0.52 | 0.56 | 0.50 | 0.54 | 0.85 | 0.54 | 0.66 | 0.67 | 0.61 | 0.65 | 0.73 | 0.72 | 0.70 | 0.66 | 0.66 |

Supplementary Table 1 shows the calculated ratio of the number of ADR reports for *older adults* (> 65 years) divided by the number of ADR reports for *younger adults* (19-65 years) per year. The calculated ratio increased slightly over the years.
