# Supplementary material for: Adverse drug reactions in older adults: a retrospective comparative analysis of spontaneous reports to the German Federal Institute for Drugs and Medical Devices
Source: BMC Pharmacol Toxicol. 2020 Mar 23;21:25. doi: 10.1186/s40360-020-0392-9 (PMC7092423; doi:10.1186/s40360-020-0392-9)
Supplement: Supplementary file 2 — Additional file 2 Supplementary Document 1. The average number of ADR reports per 100,000 inhabitants/males/females and estimation of the number of ADR reports per 100,000 assumed drug-exposed inhabitants/males/females per age group. [file 40360_2020_392_MOESM2_ESM.docx]

**Supplementary Document 1. The average number of ADR reports per 100,000 inhabitants/ males/ females and estimation of the number of ADR reports per 100,000 assumed drug-exposed inhabitants/ males/ females per age group.**

Introduction:

The exact number of drug-exposed inhabitants/ males/ females in Germany is unknown since published data of the German drug prescription reports (AVP) [24] are not patient-related. They include data on the number of prescribed drugs in DDD only, and have some further limitations like missing data on privately insured patients, over-the-counter (OTC) drug use, and lack of exact data concerning the DDD per insured males/ females. Therefore, we related the number of ADR reports to the number of German inhabitants/ males/ females per age group for each year [23] and estimated the number of assumed drug-exposed inhabitants/ males/ females based on published medication use of German younger adults in the DEGS1 study [4].

Methods:

Assumptions:

Underreporting is equally distributed by age and gender

*

*

*

*

*

*

*

1. Calculation of the number of ADR reports per 100,000 assumed drug-exposed inhabitants/ males/ females using DEGS1 data [4] (2008-2011)


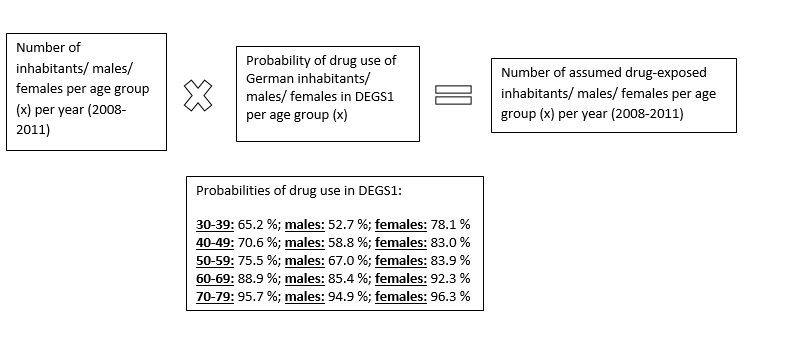


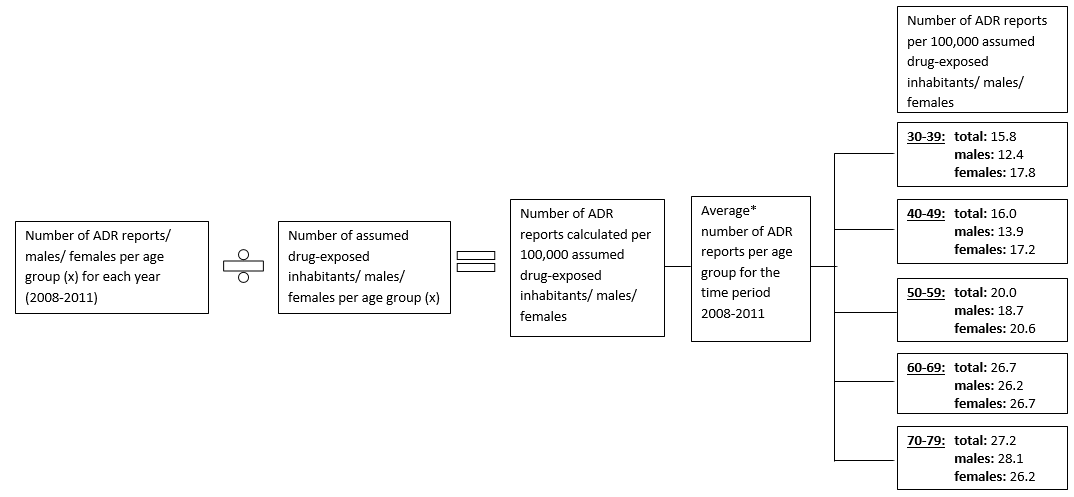


*average number of ADR reports of the four years per age group

The figure 1) shows the average number of ADR reports per 100,000 assumed drug-exposed inhabitants/ males/ females per age group. For this analysis, the time period and the age groups were adapted according to the DEGS1 analysis. All ADR reports (male, female and unknown gender) were considered for the calculation of the total average number of ADR reports per 100,000 assumed drug-exposed inhabitants. Thus, the total number of ADR reports per 100,000 assumed drug-exposed inhabitants do not lie exactly in the middle between the number of ADR reports per 100,000 assumed drug-exposed male/ female inhabitants.

Results:

- In each age group the average numbers of ADR reports per 100,000 assumed **drug-exposed inhabitants** exceeds the average numbers of ADR reports per 100,000 inhabitants (larger denominator when all inhabitants are included) (see figure 3 manuscript)
  - The greatest deviations were observed for the youngest age group 30-39 (15.8 ADR reports) with a greater deviation for males (12.4 ADR reports) due to the lower drug exposure rates in younger inhabitants, especially in younger males
  - The smallest deviation was observed for the oldest age group 70-79 (27.2 ADR reports) due to the approximation of drug exposure to 100.0 % of the older inhabitants
  - More ADR reports were observed for 70-79 year old males than females
- The average number of ADR reports per 100,000 assumed **drug-exposed inhabitants** increased with advancing age. However, the increase was slightly lower than the increase of the average number of ADR reports per 100,000 inhabitants reflecting different age and gender dependent drug-exposure rates.

Discussion:

Both analysis (average number of ADR reports per 100,000 inhabitants/ assumed **drug-exposed inhabitants**) yielded similar results. The number of ADR reports per 100,000 inhabitants and per 100,000 assumed drug-exposed inhabitants increased with rising ages. The number of ADR reports referring to males was higher in relation to 100,000 male inhabitants from the age group 66-75 onwards, and in relation to 100,000 assumed drug-exposed male inhabitants for the age group 70-79 compared to the number of ADR reports referring to females per 100,000 female inhabitants and per 100,000 assumed drug-exposed female inhabitants. The presented analysis in the manuscript regarding the observed higher increase of the number of ADR reports for older adults compared to younger adults per 100,000 inhabitants per year (Figure 2) is supported by the increase of the number of ADR reports per 100,000 inhabitants (see manuscript Figure 3) and drug-exposed inhabitants with rising age groups (in the presented supplement). The same accounts for the increase of ADR reports for *older adults* in relation to *younger adults* in the past few years (derived from the calculated ratio "number of ADR reports for *older adults*/ number of ADR reports for *younger adults*") (Additional File 1). These additional analyses support our finding that more ADR reports were seen with rising ages up to the age group 76-84 years in relation to the number of inhabitants in Figure 3.

Besides the limitations of the data sources [4, 23, 25] used, the different periods of time and age groups need to be respected when comparing the results.

Deviating from literature, we assumed that the underreporting is equally distributed by age and gender. According to literature, underreporting for males is discussed to be higher compared to females [49]. Therefore, we assume that the number of ADR reports per 100,000 males in relation to the number of inhabitants and assumed drug-exposed inhabitants could be even higher as evaluated in our analysis.
