# Supplementary material for: Adverse drug reactions in older adults: a retrospective comparative analysis of spontaneous reports to the German Federal Institute for Drugs and Medical Devices
Source: BMC Pharmacol Toxicol. 2020 Mar 23;21:25. doi: 10.1186/s40360-020-0392-9 (PMC7092423; doi:10.1186/s40360-020-0392-9)
Supplement: Supplementary file 3 — Additional file 3 Supplementary Table 2. The number of ADR reports of the potentially inappropriate medications (PIMs) contained in the PRISCUS list in older adults (> 65 years). [file 40360_2020_392_MOESM3_ESM.docx]

**Supplementary Table 2. The number of ADR reports of the potentially inappropriate medications (PIMs) contained in the PRISCUS list in *older adults* (> 65 years).**

| rank (of PIMs) | potentially inappropriate medications according to PRISCUS list [18] | number of ADR reports | % of *older adults* reports (n= 69,914) |
| --- | --- | --- | --- |
| 1. | olanzapine | 357 | 0.51 |
| 2. | etoricoxib | 294 | 0.42 |
| 3. | haloperidol | 275 | 0.39 |
| 4. | clozapine | 220 | 0.31 |
| 5. | lorazepam | 196 | 0.28 |
| 6. | amitriptyline | 132 | 0.19 |
| 7. | nitrofurantoin | 104 | 0.15 |
| 8. | sotatol | 99 | 0.14 |
| 9. | prasugrel | 95 | 0.14 |
| 10. | doxepin | 88 | 0.13 |
| 11. | zolpidem | 87 | 0.12 |
| 12. | diazepam | 80 | 0.11 |
| 13. | nifedipine | 77 | 0.11 |
| 14. | zopiclone | 73 | 0.10 |
| 15. | flecainide | 71 | 0.10 |
| 16. | trimipramine | 69 | 0.10 |
| 17. | pentoxifylline | 62 | 0.09 |
| 18. | doxazosin | 58 | 0.08 |
| 18. | clonidine | 58 | 0.08 |
| 18. | oxazepam | 58 | 0.08 |
| 19. | solifenacin | 52 | 0.07 |
| 20. | indometacin | 51 | 0.07 |
| 21. | beta-actetyldigoxin | 36 | 0.05 |
| 21. | baclofen | 36 | 0.05 |
| 22. | dimetindene | 34 | 0.05 |
| 22. | dimenhydrinate | 34 | 0.05 |
| 22. | bromazepam | 34 | 0.05 |
| 23. | piracetam | 32 | 0.05 |
| 24. | tolterodine | 31 | 0.04 |
| 24. | ticlopidine | 31 | 0.04 |
| 25. | meloxicam | 30 | 0.04 |
| 26. | oxybutynin | 28 | 0.04 |
| 26. | clomipramine | 28 | 0.04 |
| 27. | reserpine/ clopamide | 26 | 0.04 |
| 27. | naftidrofuryl | 26 | 0.04 |
| 28. | acemetacin | 25 | 0.04 |
| 28. | tranylcypromine | 25 | 0.04 |
| 29. | fluoxetine | 24 | 0.03 |
| 29. | terazosin | 24 | 0.03 |
| 30. | dimenhydrinat/ cinnarizine | 22 | 0.03 |
| 31. | tetrazepam | 21 | 0.03 |
| 32. | digoxin | 20 | 0.03 |
| 32. | clemastine | 20 | 0.03 |
| 32. | doxylamine | 20 | 0.03 |
| 33. | levomepromazine | 19 | 0.03 |
| 33. | nitrazepam | 19 | 0.03 |
| 33. | alprazolam | 19 | 0.03 |
| 34. | piroxicam | 16 | 0.02 |
| 35. | maprotiline | 15 | 0.02 |
| 36. | brotizolam | 14 | 0.02 |
| 37. | flunitrazepam | 13 | 0.02 |
| 38. | lormetazepam | 12 | 0.02 |
| 39. | fluphenazine | 11 | 0.02 |
| 40. | reserpine/ dihydralazine/ hydrochlorothaizide | 10 | 0.01 |
| 40. | dihydroergocryptine | 10 | 0.01 |
| 41. | methyldopa | 9 | 0.01 |
| 41. | clobazam | 9 | 0.01 |
| 41. | temazepam | 9 | 0.01 |
| 41. | diphenhydramine | 9 | 0.01 |
| 42. | hydroxyzine | 8 | 0.01 |
| 42. | chlorphenamin/ ascorbic acid/ paracetamol | 8 | 0.01 |
| 43. | pethidine | 7 | 0.01 |
| 43. | triazolam | 7 | 0.01 |
| 44. | ketoprofen | 6 | 0.01 |
| 44. | nitrofurantoin/ pyridoxine | 6 | 0.01 |
| 44. | perphenazine | 6 | 0.01 |
| 44. | phenobarbital | 6 | 0.01 |
| 45. | imipramine | 5 | 0.01 |
| 45. | thioridazine | 5 | 0.01 |
| 45. | chlordiazepoxide | 5 | 0.01 |
| 45. | chloral hydrate | 5 | 0.01 |
| 46. | phenylbutazone | 4 | 0.01 |
| 46. | metildigoxin | 4 | 0.01 |
| 46. | dihydroergocristine/ reserpine/ clopamide | 4 | 0.01 |
| 46. | atenolol/nifedipine | 4 | 0.01 |
| 46. | doxylamine/ dextrometorphan/ ephedrine/ paracetamol | 4 | 0.01 |
| 47. | acteyldigoxin | 3 | 0.00 |
| 47. | dihydroergocryptine/ reserpine/ clopamide | 3 | 0.00 |
| 47. | medazepam | 3 | 0.00 |
| 47. | diphenhydramine/ cyanocobalamin/ dexamethasone/ lidocaine/ pyridoxine | 3 | 0.00 |
| 48. | chlorphenamine/ codeine | 2 | 0.00 |
| 48. | triprolidine/ pseudoephedrine | 2 | 0.00 |
| 48. | prazosin | 2 | 0.00 |
| 48. | nifedipine/ metoprolol | 2 | 0.00 |
| 48. | ergotamin/ phenobarbital/ belladonna | 2 | 0.00 |
| 48. | dihydroergocryptine/ dihydroergocristine/ dihydroergocornine | 2 | 0.00 |
| 48. | paraffin liquid | 2 | 0.00 |
| 48. | diazepam/ benzoic acid/ benzyl alcohol/ propylene glycol/ sodium benzoat | 2 | 0.00 |
| 48. | prazepam | 2 | 0.00 |
| 48. | diphenhydramine/ lupus/ valariana | 2 | 0.00 |
| 48. | diphenhydramine/ carbromal | 2 | 0.00 |
| 48. | nicergoline | 2 | 0.00 |
| 48. | phenobarbital/ belladonna/ ergotamine | 2 | 0.00 |
| 48. | phenobarbital/ caffeine/ ethaverine/ paracetamol/ propylphenazone | 2 | 0.00 |
| 49. | nitrofurantoin/ phenazopyridine/ sulfadiazine | 1 | 0.00 |
| 49. | nitrofurantoin/ sulfadiazine | 1 | 0.00 |
| 49. | dimenhydrinate/ pyridoxine | 1 | 0.00 |
| 49. | reserpine/ hydrochlorothiazie | 1 | 0.00 |
| 49. | reserpine/ ajmaline/ belladonna /pentaerithrityl tetranitrate | 1 | 0.00 |
| 49. | ergotamine | 1 | 0.00 |
| 49. | ergotamine/ caffeine/ cyclizine | 1 | 0.00 |
| 49. | ergotamine/ caffeine | 1 | 0.00 |
| 49. | dihydroergotamine/ etilefrine | 1 | 0.00 |
| 49. | dihydroergotamine/ heparin/ lidocaine | 1 | 0.00 |
| 49. | dihydroergotamine/ heparin | 1 | 0.00 |
| 49. | chlordiazepoxide/ amitriptyline | 1 | 0.00 |
| 49. | chlordiazepoxide/ clidinium | 1 | 0.00 |
| 49. | flurazepam | 1 | 0.00 |
| 49. | zalpeplon | 1 | 0.00 |
| 49. | diphenhydramine/ caffeine/ polistirex | 1 | 0.00 |
| 49. | diphenhydramine/ methaqualone | 1 | 0.00 |
| 49. | diphenhydramine/ passiflora/ valeriana | 1 | 0.00 |
| 49. | phenobarbital/ acetylsalicyclic acid/ caffeine/ codeine | 1 | 0.00 |
| 49. | phenobarbital/ phenytoin | 1 | 0.00 |
| - | chinidin | 0 | 0.00 |
| - | chlorphenamin | 0 | 0.00 |
| - | triprolidine/ pseudoephedrine | 0 | 0.00 |
| - | reserpine | 0 | 0.00 |
| - | dihydroergotoxin | 0 | 0.00 |
| - | dikaliumchlorazepat | 0 | 0.00 |

Supplementary Table 2 shows the absolute and relative number of ADR reports in which one or several monosubstances and/or combination products of the PIMs contained in the PRISCUS list [18] were reported as suspected. Monosubstances and combination products are listed separately. One ADR report can contain several drug substances as suspected. Therefore, the number of drug substances may exceed the number of ADR reports.
