# Supplementary material for: Adverse drug reactions in older adults: a retrospective comparative analysis of spontaneous reports to the German Federal Institute for Drugs and Medical Devices
Source: BMC Pharmacol Toxicol. 2020 Mar 23;21:25. doi: 10.1186/s40360-020-0392-9 (PMC7092423; doi:10.1186/s40360-020-0392-9)
Supplement: Supplementary file 4 — Additional file 4 Supplementary Table 3. The 20 ADRs reported most frequently in the ADR reports of younger adults, older adults and stratified age groups. [file 40360_2020_392_MOESM4_ESM.docx]

**Supplementary Table 3. The 20 ADRs reported most frequently in the ADR reports of *younger adults*, *older adults* and stratified age groups.**

| rank | *younger adults* (19-65) (n= 111,463) % of the most frequently reported ADRs (number of reports) | OR [+/- 95 % CI] *older adults* vs. *younger adults* | *older adults* (> 65) (n= 69,914) % of the most frequently reported ADRs (number of reports) | OR [+/- 95 % CI] *older adults* vs. *younger adults* | patients aged 66-75 (n= 37,370) % of the most frequently reported ADRs (number of reports) | patients aged 76-85 (n= 24,149) % of the most frequently reported ADRs (number of reports) | OR [+/- 95 % CI] patients aged 76-85 vs. patients aged 66-75 | patients aged 86+ (n= 5,649) % of the most frequently reported ADRs (number of reports) | OR [+/- 95 % CI] patients aged ≥ 86 vs. patients aged 66-75 |
| --- | --- | --- | --- | --- | --- | --- | --- | --- | --- |
| 1. | 6.0 % (6,694) nausea | 0.9 [0.8-1.0] | 5.4 % (3,756) nausea | 0.9 [0.8-1.0] | 5.9 % (2,206) nausea | 5.1 % (1,228) nausea | 0.9 [0.8-1.0] | 5.3 % (299)  death ^a^ | 3.9 [3.0-4.9]* |
| 2. | 4.4 % (4,959) dizziness | 1.0 [0.9-1.1] | 4.5 % (3,177) dizziness | 1.0 [0.9-1.1] | 4.6 % (1,731) dizziness | 4.8 % (1,148) dizziness | 1.0 [0.9-1.2] | 4.3 % (244) gastrointestinal haemorrhage | 3.3 [2.6-4.3]* |
| 3. | 4.2 % (4,678) dyspnoea | 1.0 [0.9-1.0] | 4.0 % (2,821) dyspnoea | 1.0 [0.9-1.0] | 4.4 %(1,641) dyspnoea | 3.7 % (884) dyspnoea | 0.8 [0.7-0.9]* | 3.9 % (223)  nausea | 0.7 [0.5-0.8]* |
| 4. | 4.0 % (4,421) pruritus | 0.8 [0.7-0.9]* | 3.4 % (2,404) diarrhoea | 1.1 [1.0-1.2] | 3.7 % (1,401) diarrhoea | 3.1 % (754) vomiting | 1.0 [0.9-1.2] | 3.8 % (214) dizziness | 0.8 [0.6-1.0] |
| 5. | 3.4 % (3,786)  rash | 0.8 [0.7-0.8]* | 3.2 % (2,265) pruritus | 0.8 [0.7-0.9]* | 3.7 % (1,367) pruritus | 3.1 % (745) diarrhoea | 0.8 [0.7-0.9]* | 3.2 % (180) diarrhoea | 0.8 [0.7-1.1] |
| 6. | 3.3 % (3,673) headache | 0.6 [0.5-0.7]* | 3.1 % (2,142) vomiting | 1.1 [1.0-1.2] | 3.1 % (1,168) vomiting | 3.0 % (719)  pruritus | 0.8 [0.7-0.9]* | 3.2 % (178)  fall | 2.8 [2.1-3.7]* |
| 7. | 3.1 % (3,432) diarrhoea | 1.1 [1.0-1.2] | 2.6 % (1,808)  rash | 0.8 [0.7-0.8]* | 3.0 (1,122) rash | 2.7 % (648)  death ^a^ | 1.9 [1.6-2.3]* | 3.1 % (174) vomiting | 1.0 [0.8-1.3] |
| 8. | 2.9 % (3,238) vomiting | 1.1 [1.0-1.2] | 2.3 % (1,595) thrombocytopenia | 1.3 [1.2-1.5]* | 2.6 % (973) erythema | 2.5 % (610) hypertension | 1.3 [1.1-1.5]* | 2.9 % (161) cerebrovascular accident | 2.4 [1.8-3.2]* |
| 9. | 2.8 % (3,146) erythema | 0.8 [0.7-0.8]* | 2.3 % (1,581)  death ^a^ | 3.8 [3.3-4.4]* | 2.4 % (909) myalgia | 2.3 % (566)  rash | 0.8 [0.7-0.9]* | 2.8 % (157) cerebral infarction | 2.8 [2.1-3.9]* |
| 10. | 2.7 % (3,033) fatigue | 0.8 [0.7-0.9]* | 2.2 % (1,543) hypertension | 1.6 [1.5-1.8]* | 2.4 % (903) headache | 2.3 % (563) gastrointestinal haemorrhage | 1.8 [1.4-2.2]* | 2.7 % (154) dyspnoea | 0.6 [0.5-0.8]* |
| 11. | 2.6 % (2,855) hypersensitivity | 0.6 [0.6-0.7]* | 2.2 % (1,518) fatigue | 0.8 [0.7-0.9]* | 2.3 % (878) fatigue | 2.3 % (562) thrombocytopenia | 1.0 [0.8-1.2] | 2.5 % (141) cerebral haemorrhage | 2.6 [1.9-3.6]* |
| 12. | 2.4 % (2,681) urticaria | 0.5 [0.4-0.5]* | 2.2 % (1,518) erythema | 0.8 [0.7-0.8]* | 2.3 % (874) thrombocytopenia | 2.2 % (534) fall | 1.9 [1.6-2.4]* | 2.5 % (141) haemoglobin decreased | 2.3 [1.7-3.2]* |
| 13. | 2.2 % (2,507) pyrexia | 0.8 [0.7-0.9]* | 2.0 % (1,421) headache | 0.6 [0.5-0.7]* | 2.1 % (801) pyrexia | 2.1 % (511) cerebrovascular accident | 1.8 [1.4-2.2]* | 2.5 % (139) hypertension | 1.2 [0.9-1.7] |
| 14. | 2.2 % (2,503) myalgia | 0.8 [0.8-0.9]* | 2.0 % (1,405) asthenia | 1.3 [1.2-1.5]* | 2.0 % (755) hypersensitivity | 2.0 % (490) asthenia | 1.0 [0.9-1.2] | 2.3 % (131) asthenia | 1.2 [0.9-1.6] |
| 15. | 2.0 % (2,259)  drug ineffective | 0.8 [0.7-0.9]* | 1.9 % (1,343) gastrointestinal haemorrhage | 5.1 [4.2-6.1]* | 2.0 % (754) hypertension | 2.0 % (473) cerebral infarction | 2.0 [1.6-2.5]* | 2.3 % (130)  pruritus | 0.6 [0.5-0.8]* |
| 16. | 1.8 % (2,047) tachycardia | 0.7 [0.6-0.8]* | 1.9 % (1,334) myalgia | 0.8 [0.8-0.9]* | 2.0 % (736) asthenia | 1.9 % (458) fatigue | 0.8 [0.7-1.0] | 2.2 % (125) confusional state | 2.2 [1.6-3.0]* |
| 17. | 1.7 % (1,915) thrombocytopenia | 1.3 [1.2-1.5]* | 1.8 % (1,248) pyrexia | 0.8 [0.7-0.9]* | 1.7 % (631) drug ineffective | 1.9 % (458) syncope | 1.3 [1.1-1.6]* | 2.2 % (122) cardiac failure | 2.0 [1.4-2.7]* |
| 18. | 1.7 % (1,914) hepatic enzyme increased | 0.6 [0.5-0.7]* | 1.7 % (1,219) fall | 3.0 [2.6-3.6]* | 1.6 % (595) hyperhidrosis | 1.9 % (453) erythema | 0.7 [0.6-0.9]* | 2.2 % (122) hypoglycaemia | 3.2 [2.3-4.6]* |
| 19. | 1.7 % (1,888) paraesthesia | 0.5 [0.4-0.6]* | 1.7 % (1,187) cerebrovascular accident | 3.0 [2.6-3.6]* | 1.6 % (580) arthralgia | 1.9 % (449) cerebral haemorrhage | 1.9 [1.5-2.5]* | 2.2 % (122)  syncope | 1.5 [1.1-2.1]* |
| 20. | 1.7 % (1,865) arthralgia | 0.8 [0.7-0.9]* | 1.6 % (1,151) syncope | 1.7 [1.5-2.0]* | 1.5 % (560) acute kidney injury | 1.8 % (441) heamoglobin decreased | 1.7 [1.3-2.1]* | 2.1 % (119) melaena | 2.9 [2.0-4.1]* |

*OR=1 is not included; OR > 1 reported more often in *older adults,* patients aged 66-75; OR < 1 reported more often in *younger adults* or the respective age groups

^a^ except for ADRs also diagnosis, results of investigations, or social histories can be coded according to MedDRA terminology [25]. The preferred term (PT) "death" itself is not an ADR but an outcome coded by MedDRA terminology [25].

Supplementary Table 3 shows the relative and absolute numbers of the 20 ADRs reported most frequently in the ADR reports of *older adults*, *younger adults* and the stratified age groups with the calculated odds ratios with Bonferroni adjusted confidence intervals. The dataset *younger adults* served as a reference for the calculation of the odds ratios for *older adults* vs. *younger adults*. The dataset patients aged 66-75 years served as a reference for the calculation of the odds ratios for patients aged 76-85 years and patients aged ≥ 86 years vs. patients aged 66-75 years. The ADR evaluation refers to the PT-level of MedDRA terminology [25]. One ADR report can inform about several ADRs. Therefore, the number of ADRs exceeds the number of ADR reports.

Interestingly, allergic-type reactions like "erythema", "pruritus", "rash", and "hypersensitivity" were reported more often for *younger adults* than for *older adults*. This may be explained by differences with regard to (i) the used drugs between older and younger adults, (ii) an overrepresentation of other, non-allergic type ADRs in older adults, or (iii) differences in immunological response between older and younger adults. Further research is needed to evaluate if older adults are less prone to develop allergic-type reactions than younger adults.
