# Supplementary material for: Adverse drug reactions in older adults: a retrospective comparative analysis of spontaneous reports to the German Federal Institute for Drugs and Medical Devices
Source: BMC Pharmacol Toxicol. 2020 Mar 23;21:25. doi: 10.1186/s40360-020-0392-9 (PMC7092423; doi:10.1186/s40360-020-0392-9)
Supplement: Supplementary file 5 — Additional file 5 Supplementary Table 4. The three drug substances most frequently suspected for the three most frequently reported ADRs in the ADR reports of antithrombotic agents of younger adults and older adults. [file 40360_2020_392_MOESM5_ESM.docx]

**Supplementary Table 4. The three drug substances most frequently suspected for the three most frequently reported ADRs in the ADR reports of antithrombotic agents of *younger adults* and *older adults*.**

| rank | *younger adults* (19-65) the three most frequently reported ADRs in the ADR reports of antithrombotic agents (n= 5,676) | the three most frequently reported drug substances (number of reports) per ADR | rank | *older adults* (> 65) the three most frequently reported ADRs in the ADR reports of antithrombotic agents (n= 13,831) | the three most frequently reported drug substances (number of reports) per ADR |
| --- | --- | --- | --- | --- | --- |
| 1. | 6.5 % (367) thrombocytopenia | 44.9 % (165) heparin  15.8 % (58) tirofiban  8.2 % (30) clopidogrel  8.2 % (30) enoxaparin | **1.** | 7.6 % (1,051) gastrointestinal haemorrhage | 26.9 % (283) acetylsalicyclic acid  23.0 % (242) rivaroxaban  21.6 % (227) phenprocoumon |
| 2. | 6.3 % (358) pulmonary embolism | 29.6 % (106) certoparin  21.5 % (77) enoxaparin  15.9 % (57) rivaroxaban | **2.** | 5.9 % (812) cerebral haemorrhage | 35.2 % (286) rivaroxaban  14.8 % (120) apixaban  13.4 % (109) phenprocoumon |
| 3. | 3.7 % (211) haemorrhage | 19.9 % (42) rivaroxaban  17.0 % (36) enoxaparin  11.4 % (24) phenprocoumon | **3.** | 4.9 % (677) haemorrhage | 30.2 % (205) rivaroxaban  17.6 % (119) apixaban  13.7 % (93) dabigatran |

Supplementary Table 4 shows the relative and absolute numbers of the three drug substances most frequently reported as suspected in the ADR reports of antithrombotic agents of *younger adults* (19-65) and *older adults* (> 65). One ADR report can contain several drug substances as suspected. Therefore, the number of drug substances exceeds the number of ADR reports.
