# Supplementary material for: Adverse drug reactions in older adults: a retrospective comparative analysis of spontaneous reports to the German Federal Institute for Drugs and Medical Devices
Source: BMC Pharmacol Toxicol. 2020 Mar 23;21:25. doi: 10.1186/s40360-020-0392-9 (PMC7092423; doi:10.1186/s40360-020-0392-9)
Supplement: Supplementary file 6 — Additional file 6 Supplementary Table 5. Characteristics, drug indications, and ADRs in the ADR reports of younger adults and older adults in which rivaroxaban was suspected before and after extension of the indication (01/13/2012). [file 40360_2020_392_MOESM6_ESM.docx]

**Supplementary Table 5. Characteristics, drug indications, and ADRs in the ADR reports of *younger adults* and *older adults* in which rivaroxaban was suspected before and after extension of the indication (13.01.2012).**

|  | **receipt date before 13.01.2012** | | **receipt data after 13.01.2012 (incl. 13.01.2012)** | |
| --- | --- | --- | --- | --- |
|  | ***younger adults* (19-65)** | ***older adults* (> 65)** | ***younger adults* (19-65)** | ***older adults* (> 65)** |
| **number of reports** | 73 | 138 | 1,100 | 4,287 |
| **mean age (median) [yr]** | 55.8 (59.0) | 75.3 (75.0) | 52.4 (55.0) | 78.6 (78.0) |
| **female/**  **male/**  **unknown** | 45.2 % (33)  53.4 % (39)  1.4 % (1) | 65.2 % (90)  34.8 % (48)  0 % (0) | 49.5 % (545)  49.6 % (546)  0.8 % (9) | 50.8 % (2,179)  47.9 % (2,055)  1.2 % (53) |
| **the 5 most frequently reported indication terms** | | | | |
| **1.** | 41.1 % (30) thrombosis prophylaxis | 57.2 % (79) thrombosis prophylaxis | 27.0 % (297) venous thromboembolism | 57.7 % (2,473) atrial fibrillation |
| **2.** | 15.1 % (11) venous thrombolism | 11.6 % (16) total knee replacement | 23.7 % (261) cerebrovascular accident prophylaxis | 52.6 % (2,257) cerebrovascular accident prophylaxis |
| **3.** | 12.3 % (9) total knee replacement | 10.1 % (14) knee arthroplasty | 23.0 % (253) atrial fibrillation | 14.0 % (600) unknown |
| **4.** | 8.2 % (6) unknown | 6.5 % (9) hip arthroplasty | 17.0 % (187) deep vein thrombosis | 7.8 % (336) venous thromboembolism |
| **5.** | 5.5 % (4) total hip replacement | 5.1 % (7) total hip replacement | 16.7 % (184) pulmonary embolism | 5.2 % (222) pulmonary embolism |
| **the 5 most frequently reported ADRs** | | | | |
| **1.** | 21.9 % (16) deep vein thrombosis | 18.1 % (25) deep vein thrombosis | 9.2 % (101) menorrhagia | 8.0 % (343) epistaxis |
| **2.** | 13.7 % (10) haematoma | 13.0 % (18) pulmonary embolism | 5.2 % (57) dizziness | 7.1 % (305) cerebral haemorrhage |
| **3.** | 11.0 % (8) pheripheral swelling | 7.2 % (10) dyspnea  7.2 % (10) haematoma  7.2 % (10) thrombosis | 4.8 % (53) pulmonary embolism | 5.8 % (249) haemoglobin decreased |
| **4.** | 9.6 % (7) pulmonary embolism |  | 4.7 % (52) drug ineffective | 5.8 % (247) gastrointestinal haemorrhage |
| **5.** | 8.2 % (6) haemarthrosis |  | 4.5 % (49) deep vein thrombosis | 5.1 % (217) haemorrhage |

Supplementary Table 5 shows the characteristics, reported drug indication terms, and reported ADRs in the ADR reports of *younger adults* and *older adults* in which rivaroxaban was reported as suspected drug substance before and after the extension of the indication (13.01.2012). One report may inform about more than one drug indication term and several ADRs. Therefore, the number of indication terms and ADRs exceeds the number of reports.
