# Supplementary material for: Adverse drug reactions in older adults: a retrospective comparative analysis of spontaneous reports to the German Federal Institute for Drugs and Medical Devices
Source: BMC Pharmacol Toxicol. 2020 Mar 23;21:25. doi: 10.1186/s40360-020-0392-9 (PMC7092423; doi:10.1186/s40360-020-0392-9)
Supplement: Supplementary file 7 — Additional file 7 Supplementary Table 6. The five most frequently reported ADRs of younger adults in which phenprocoumon, acytylsalicyclic acid, and apixaban were reported as suspected drug substance. [file 40360_2020_392_MOESM7_ESM.docx]

**Supplementary Table 6. The five most frequently reported ADRs of *younger adults* in which phenprocoumon, acytylsalicyclic acid, and apixaban were reported as suspected drug substance.**

| rank | phenprocoumon (n= 768; 0.7 %) | rank | acetylsalicyclic acid (n= 736; 0.7 %) | rank | apixaban (n= 293; 0.3 %) |
| --- | --- | --- | --- | --- | --- |
| 1. | 10.5 % (81) prothrombin time prolonged | **1.** | 13.6 % (100) gastrointestinal haemorrhage | **1.** | 4.8 % (14) cerebrovascular accident |
| 2. | 7.4 % (57) gastrointestinal haemorrhage | **2.** | 12.0 % (88) melaena | **1.** | 4.8 % (14) off label use |
| 3. | 6.8 % (52) drug interaction | **3.** | 7.9 % (58) nausea | **1.** | 4.8 % (14) pulmonary embolism |
| 3. | 6.8 % (52) hepatic enzyme increased | **4.** | 7.9 % (58) gastric ulcer haemorrhage | **2.** | 4.4 % (13) nausea |
| 4. | 5.9 % (45) international normalised ratio increased | **5.** | 7.2 % (53) abdominal pain upper  7.2 % (53) vomiting | **3.** | 4.1 % (12) dizziness |

Supplementary Table 6 shows the five most frequently reported ADRs in the ADR reports of *younger adults* (19-65) (n= 111,463) in which phenprocoumon, acetylsalicyclic acid, and apixaban were reported as suspected drug substance. One ADR report may inform about several ADRs. Therefore, the number of ADRs exceeds the number of ADR reports.
